# Prevalence of oral mucosal lesions in patients with systemic Lupus Erythematosus: a systematic review and meta-analysis

**DOI:** 10.1186/s12903-023-03783-5

**Published:** 2023-12-21

**Authors:** Fei Du, Wanying Qian, Xinna Zhang, Le Zhang, Jianwei Shang

**Affiliations:** 1 Department of Oral Pathology, School of Medicine, Tianjin Stomatological Hospital, Nankai University, No. 75 Dagu North Rd, Tianjin, 300041 China; 2 Tianjin Key Laboratory of Oral and Maxillofacial Function Reconstruction, Tianjin, 300041 China

**Keywords:** Lupus Erythematosus, systemic, Oral mucosal lesions, Prevalence, Meta-analysis

## Abstract

**Background:**

Systemic lupus erythematosus (SLE) is a chronic autoimmune disease that can cause a range of symptoms, including oral mucosal lesions (OMLs). The prevalence of OMLs in SLE patients and their associated factors have been studied in various regions, but the results are inconsistent. This study aims to evaluate the prevalence of OMLs in patients with SLE.

**Methods:**

Observational studies of OML prevalence in SLE patients published before 2022 were retrieved from PubMed, Embase, Web of Science, Google Scholar, and the Cochrane Library without language restriction. The quality of the studies was assessed using the Newcastle-Ottawa Scale (NOS) and Agency for Healthcare Research and Quality (AHRQ).

**Results:**

Our meta-analysis included 113 studies with a total of 53,307 SLE patients. We found that the prevalence of OMLs in SLE patients was 31% (95% CI: 28%, 35%), with oral ulcers being present in 30% of SLE patients (95% CI: 26%, 33%). Subgroup analysis showed that the prevalence of OMLs varied significantly by region, disease activity, and sample size (*p* ≤ 0.01). However, gender and year of publication had little effect on the prevalence of OMLs (*p* = 0.78 and 0.30, respectively). Oral ulcers were significantly associated with age of onset (*p* = 0.02), geographic location (*p* < 0.01), and race (*p* < 0.01). We also found that the prevalence of oral erythema was 9%, oral candidiasis was 9%, petechiae was 8%, cheilitis was 6%, and white plaque was 3%.

**Conclusions:**

Our analysis showed that the prevalence of OMLs varied significantly by region and disease activity, and child-onset patients of Indian, Malay, and Caucasian descent were more likely to have oral ulcers. The high prevalence of OML in SLE patients emphasizes the importance of regular oral examination and management in the comprehensive care of individuals with SLE.

**Supplementary Information:**

The online version contains supplementary material available at 10.1186/s12903-023-03783-5.

## Background

Systemic lupus erythematosus (SLE) is a chronic autoimmune disease with a poorly understood pathogenesis [1]. Genetic susceptibility, environmental triggers, and hormonal and sociodemographic variables may contribute to the onset and progression of SLE [2]. The global prevalence of SLE varies between 13 and 7713.5 per 100,000 person-years [3].

Clinically, SLE is highly heterogeneous and its symptoms can be influenced by age. SLE is more common in women of reproductive age but can develop at any age, with 10–20% of cases occurring in children and adolescents under 18 [4]. It was reported that juvenile lupus erythematosus is far more severe than the adult variant [5]. Up to ten years after the first diagnosis, dizziness, and new system/organ involvement may emerge after a protracted period of remission [5]. Late-onset SLE, which starts after 50 years of age, accounts for 2–20% of all SLE cases [6, 7]. Late-onset lupus typically has a gradual onset and mild disease activity [8]. In addition, several studies have uncovered variations in the clinical presentation and prevalence of SLE among populations of distinct geographic and ethnic origins. According to the studies by Izmirly et al. [9] and Al-Arfaj et al. [10], SLE is found in 72.8 per 100,000 people in the United States and in 19.28 per 100,000 people in Saudi Arabia. In contrast, Africa and Ukraine have an SLE incidence of 0.3/100,000 person-years [11, 12]. This clinical heterogeneity poses various challenges in the clinical diagnosis and treatment of SLE.

SLE can involve multiple organs and systems, including the skin, joints, kidneys, lungs, and central nervous system [13]. Oral manifestations of SLE are also typical and may include oral ulcers, honeycomb plaques, raised keratotic plaques, nonspecific erythema, purpura, petechiae, and cheilitis [14]. Oral ulcers are the most prevalent symptom of oral presentations, which manifest as single or multiple pale yellow and grayish-white superficial ulcers on the lips, cheeks, tongue, or palate, and are accompanied by pain when irritated. Oral mucosal lesions (OMLs) make food consumption difficult for patients and serve as a portal for bacterial invasion. Consequently, prevention and treatment of OMLs are essential.

Oral lesions in SLE patients are well characterized clinically and histologically, but the rate of mucosal involvement in SLE patients is still a matter of debate [15]. This systematic review and meta-analysis were conducted to evaluate the prevalence of OMLs in SLE patients by reviewing published studies on oral mucosal involvement in SLE worldwide, and to explore the difference in the prevalence of OMLs among various subgroups of SLE patients.

## Methods

This meta-analysis was conducted following the Preferred Reporting Items for Systematic Reviews and Meta-Analyses (PRISMA) statement [16] (PROSPERO registration number: CRD42022307095) [17]. Since this is a meta-analysis of previously published studies, neither ethical approval nor patient consent was required.

### Search strategy and study selection

Studies of OML prevalence in SLE patients were searched and retrieved from PubMed, Embase, Web of Science, Google Scholar, and the Cochrane Library using the search terms “Lupus Erythematosus, Systemic”, “Mouth Mucosa”, “Oral Manifestations”, “Prevalence”, and “Epidemiology”. The comprehensive search strategy for each database is shown in Supplementary Table S1.

Publications that met the PECOS criteria were included in this systematic review: Population: Males and females without age restrictions; Exposure: Systemic lupus erythematosus (diagnosed using any recognized diagnostic criteria); Comparator: None; Outcome: Prevalence of oral mucosal involvement; and Study design: Observational studies published before January 2022. Exclusion criteria: (1) Participants with other autoimmune diseases (such as pemphigoid, lichen planus, Sjögren’s syndrome, or pemphigus); (2) Case reports, conference abstracts, reviews, and meta-analysis; (3) Studies with uncertainty in the prevalence of oral mucosal illness.

Duplicate records were first identified and removed, and articles were then independently selected by two reviewers based on the abstracts and titles retrieved from the databases. Studies that did not meet the inclusion criteria were omitted. The eligibility of full-text publications was evaluated based on the selection criteria. A third reviewer was consulted in cases of disagreement.

### Data extraction and quality assessment

Data were extracted from the selected studies by two researchers independently, including authors, year of publication, country/geographic location, study design, sample size, age, gender, prevalence of overall and partial oral lesions, diagnostic criteria, and main conclusions. Incompletely revealed data were calculated and aggregated when necessary [18].

The quality of case-control and cohort studies was evaluated using the Newcastle-Ottawa Scale (NOS), which consists of three factors, namely selection, comparability, and outcome. If a study scores less than 5, it is deemed to be of poor quality. Cross-sectional studies were assessed by the 11-item Agency for Healthcare Research and Quality (AHRQ) scale. A score of 0–3 indicates low quality, a score of 4–7 indicates medium quality, and a score of 8–11 indicates good quality. The consensus score was determined using the following: (1) If the scores of raters 1 and 2 are identical, the score is used as the consensus score; (2) If the scores of raters 1 and 2 are not equal, a consensus score is determined through discussion; (3) If no consensus is reached after discussion, the consensus score is determined by a third rater, and all three raters must agree on the final judgment [19].

### Statistical analysis

Meta-analysis was performed using R4.0.2. Data with non-normal distribution are modified to comply with or approach normal distribution to increase the reliability of the pooled results. Heterogeneity was analyzed using the *I*^*2*^ statistic [20]. When heterogeneity (*P* < 0.1 (Q test) and *I*^*2*^ > 50%) is present, a random effects model [21] was used; otherwise, a fixed effects model [22] was used. The source(s) of heterogeneity and differences in the prevalence of oral mucosal disorders among different groups were determined by subgroup analysis. The effect of each included study on the overall effect size was ascertained by sensitivity analysis. Publication bias was assessed using a funnel plot, and asymmetry in the funnel plot was determined using Egger’s test [23]. A *P* < 0.05 was considered statistically significant.

## Results

### Literature search results

**Fig. 1:**
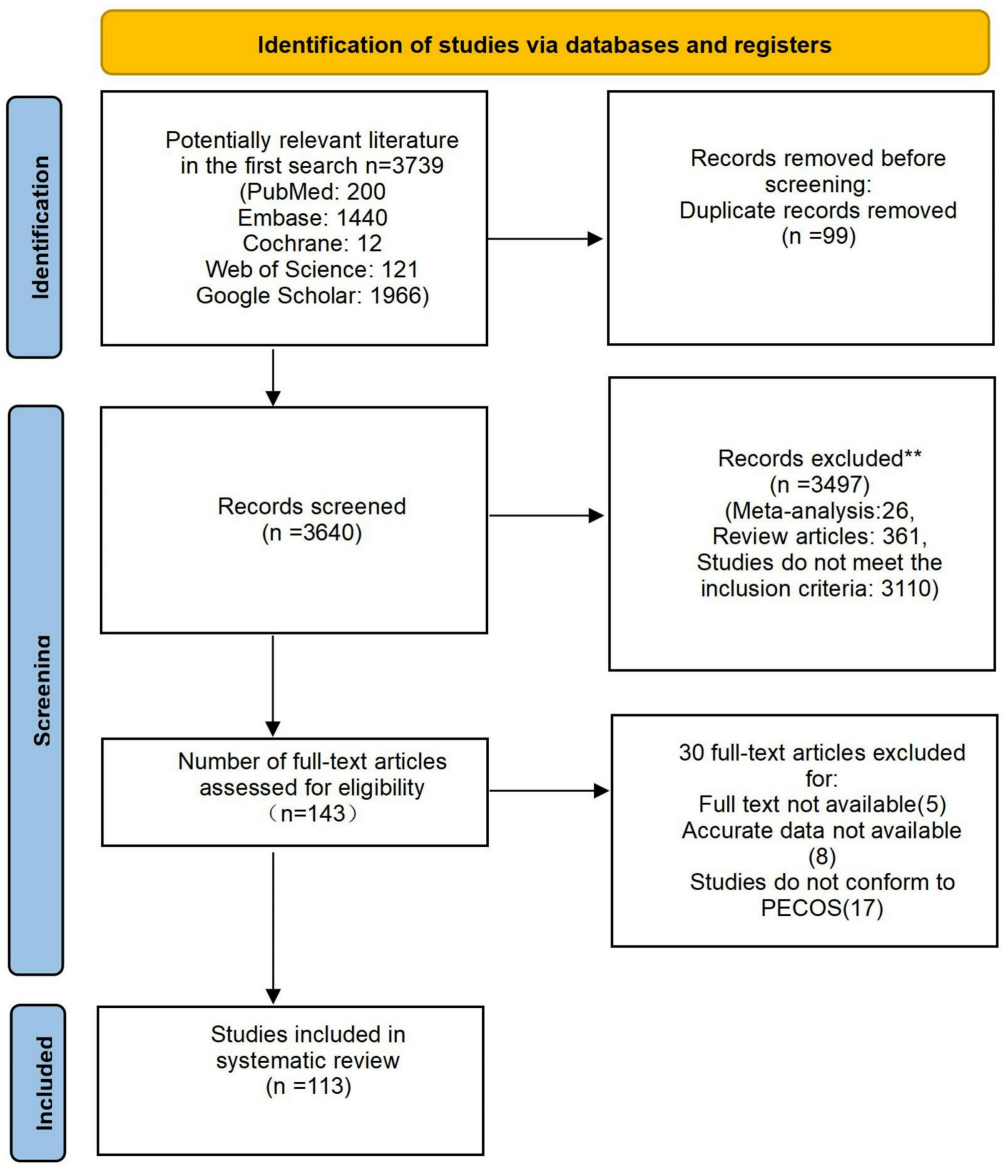
flow diagram summarizing the study selection process

The initial search yielded 3,739 articles, of which 99 duplicate records were removed. We examined the titles and abstracts of 3,640 publications and excluded 26 meta-analyses and 361 reviews. A total of 3,110 articles were excluded because they did not meet the inclusion criteria, leaving 143 articles for full-text screening. We further eliminated 17 articles that did not conform to the PECOS criteria, 8 articles with ambiguous data, and 5 articles for which the full text was not available. A final total of 113 publications were included in this systematic review and meta-analysis. Figure 1 shows the PRISMA flowchart for study selection.

### Study characteristics

Supplementary Table S2 summarizes the characteristics of the included studies. We identified 113 studies that met the inclusion criteria, among which 57 were conducted in various regions of Asia, including East Asia (n = 19), South Asia (n = 12), West Asia (n = 19), and Southeast Asia (n = 7). Additionally, 28 studies were conducted in Europe, 13 in South America, 4 in North America, 1 in Oceania, and 9 in Africa. One study by Johnson included a sample population from both South America and Europe. This systematic review involved 53,307 participants, ranging from 8 to 5,645 per study. Age was reported in median (and/or range) in 17 studies (median age 10.8–48 years) and in mean (range) or mean (SD) in 70 studies (mean age 10.892–55.4 years). Twelve studies provided no age information, while fourteen studies documented the prevalence of oral mucosal disease in SLE patients of various ages. Fifteen studies separately described the prevalence of oral mucosal disease in SLE patients of different genders. Our meta-analysis comprised a total of 113 articles, of which only 109 articles reported on the overall prevalence of oral diseases. However, we also included four additional articles that reported on the prevalence of specific oral diseases, such as oral ulcers, in our overall review. Of the 113 articles, 111 studies examined the prevalence of oral ulcers among patients with SLE. Furthermore, our analysis also investigated the prevalence of various types of oral lesions, such as central erythema with white speckles or striae in two articles, erythema in five articles, white plaque in four articles, oral candidiasis in five articles, petechiae in four articles, and cheilitis in three articles.

For the diagnosis of SLE, all studies except five [24–28] met the American College of Rheumatology (ACR) 1997/1982 [29, 30] and/or Systemic Lupus International Collaborating Clinics (SLICC) 2012 [31] classification criteria for SLE or were based on clinical examination by a qualified physician or rheumatologist.

### Methodological quality

Cross-sectional studies were evaluated using the AHRQ scale, cohort and case-control studies were assessed by the NOS scale. Our results revealed that the overall quality of the included studies was high, with 93 studies of moderate quality, 18 of high quality, and 2 of poor quality. The included articles consisted of 43 cross-sectional, 64 cohort, and 6 case-control studies. The results of the methodological quality evaluation are summarized in Supplementary Table S2.

### Meta-Analysis

#### Overall prevalence of OMLs in SLE patients

##### Overall prevalence

**Fig. 2:**
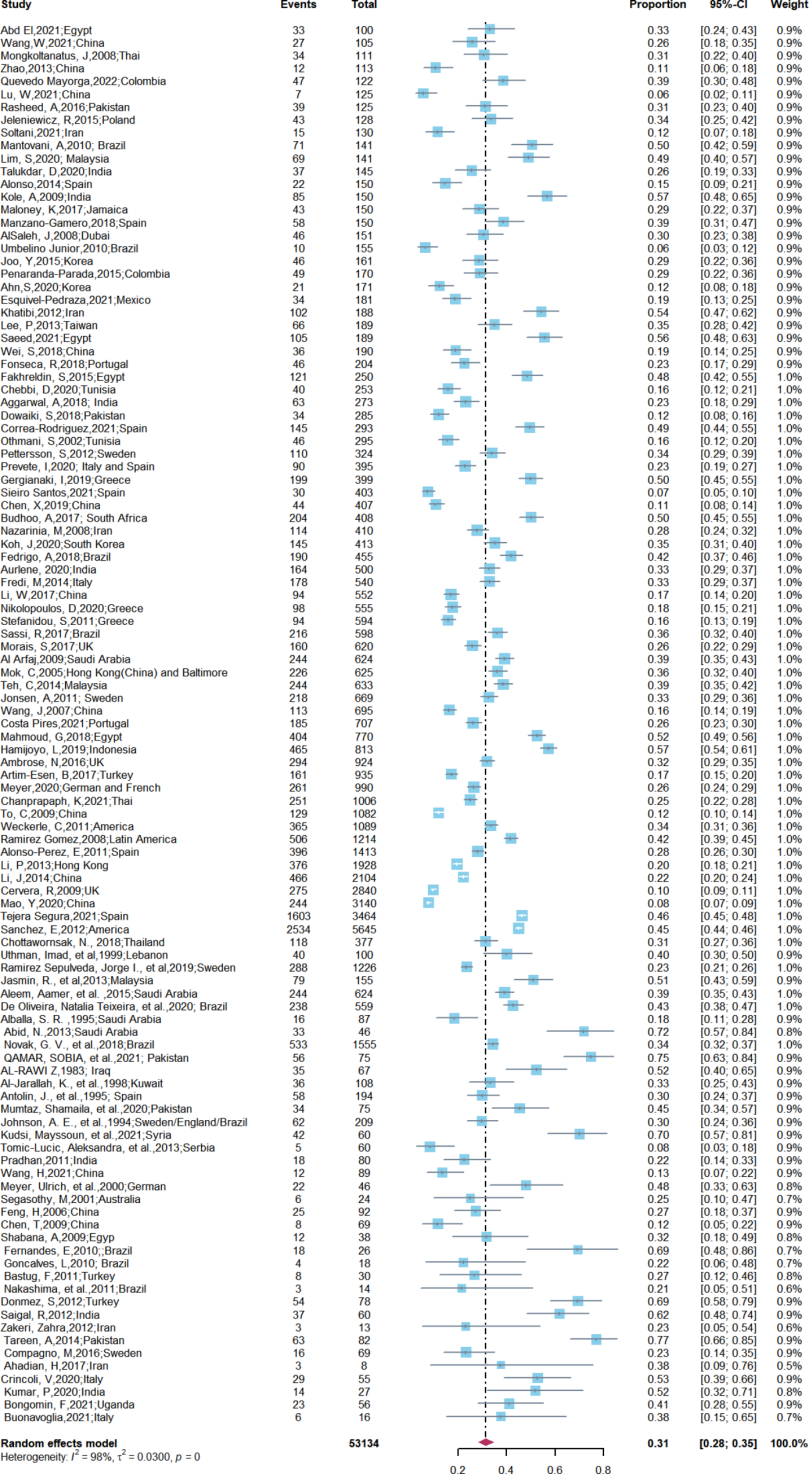
Prevalence of oral mucosal lesions among SLE patients (Forest plot of the included studies)

A total of 109 studies were included in the meta-analysis of the prevalence of oral mucosal disease in SLE patients. A random effects model was used due to high heterogeneity among studies (*I*^*2*^ = 98%, τ^2^ = 0.0300, *P* = 0). Our findings demonstrated that the overall prevalence of oral mucosal illness among SLE patients was 31% (95% CI: 28–35%) (Fig. 2).

##### Subgroup analysis of OML prevalence in SLE patients

Subgroup analyses of the prevalence of OMLs in SLE patients by gender, year of publication, region, disease state, and sample size are summarized in Supplementary Table S3.

##### Gender subgroups

The prevalence of OMLs was 34% in male SLE patients and 37% in female SLE patients, with no significant difference between gender (*P* = 0.78). (Supplementary Figure S2)

##### Year of publication subgroups

The prevalence of OMLs in SLE patients was 35% before 2000, 26% between 2000 and 2009, 35% between 2010 and 2015, and 31% between 2016 and 2022. These differences were not statistically significant (*P* = 0.30). (Supplementary Figure S3)

##### Region subgroups

South Asia had the highest prevalence (42%) of OMLs in SLE patients, followed by Southeast Asia (40%), West Asia (39%), Africa (37%), South America (34%), North America (31%), and Europe (28%). In contrast, OML prevalence was lowest in East Asia (18%), with an intermediate prevalence of 25% observed in Oceania. The difference in OML prevalence across regions was statistically significant (*P* < 0.01). (Supplementary Figure S4)

##### Disease state subgroups

OMLs were significantly more prevalent in active SLE (77%) than in inactive SLE (18%) (*P* < 0.01). (Supplementary Figure S5)

##### Sample size

Of the 109 studies reviewed, 81 had a sample size of over 100, with a synthetic OML prevalence rate of 29%, significantly lower than the rate of 40% in the remaining 28 studies with a sample size below 100. (*P* = 0.01) (Supplementary Figure S6).

##### Sensitivity analysis

We performed a sensitivity analysis of the total OML prevalence by excluding studies individually. We found that the removal of different studies resulted in a comparable total detection rate, demonstrating that the results of this study were stable. (Supplementary Figure S7)

##### Publication Bias

We generated a funnel plot to assess publication bias. In line with Egger’s test results (t = 0.89, df = 107, *P* = 0.3734), the funnel plot of the total prevalence of OMLs in SLE patients did not reveal marked asymmetry, indicating that there was no significant publication bias (Supplementary Figure S8).

#### Oral ulcers prevalence in SLE patients

##### Oral ulcers prevalence

Meta-analysis of the 111 studies that reported oral ulcers yielded a 30% overall prevalence of oral ulcer (95% CI: 26–33%; heterogeneity: *I*^*2*^ = 98%, *P* < 0.01; 53,124 patients), one of the studies encompassed samples from two distinct regions, namely Europe and South America. (Supplementary Figure S1)

##### Subgroup analysis of oral ulcers prevalence in SLE patients

Subgroup analysis revealed significant differences in the prevalence of oral ulcers among SLE patients of different regions, ethnicities, and ages. Please refer to Supplementary Table S4.

##### Age of onset subgroups

Oral ulcers were more prevalent in patients with childhood-onset SLE (41%) than in those with adult-onset (26%) and late-onset (26%) SLE. The differences were statistically significant (*P* = 0.02). (Supplementary Figure S9)

##### Race subgroups

There were significant differences in the prevalence of oral ulcers among different race groups (*P* < 0.01). Indian SLE patients had the highest prevalence of oral ulcers (56%), followed by Malays (44%), Caucasians (41%), and Africans (33%). East Asians had the lowest prevalence of oral ulcer at 29%. (Supplementary Figure S10)

##### Region subgroups

Among SLE patients, the prevalence of oral ulcer was found to be highest in South Asia (42%) and Southeast Asia (40%), followed by South America (35%), Africa (33%), West Asia (33%), Europe (27%), Oceania (25%), East Asia (18%), and North America (16%). (*P* < 0.01) (Supplementary Figure S11).

#### Erythema

A meta-analysis of 5 studies showed that the prevalence of Erythema in SLE patients was 9% (95% CI: 5—14%; heterogeneity: *I*^*2*^ = 66%, *P* = 0.02; 530 patients). Supplementary Figure S12 provides a visual representation of the data. Notably, the study conducted by Meyer [32] had results that were well beyond the range of the other investigations, which may have contributed to the heterogeneity. After excluding this study, the results indicated a prevalence of Erythema of 7% (95% CI: 4—11%; heterogeneity: *I*^*2*^ = 47%, *P* = 0.13).

#### White plaque

A meta-analysis of four studies showed that the prevalence of white plaque was 3% (95% CI: 1–5%; heterogeneity: *I*^*2*^ = 0%, *P* = 0.84; 457 patients). (Supplementary Figure S13)

#### Oral candidiasis

Based on the analysis of five studies, the prevalence of oral candidiasis was found to be 9% (95% CI: 1—25%; heterogeneity: *I*^*2*^ = 96%, *P* < 0.01; 469 patients), as presented in Supplementary Figure S14. It is worth noting that the study conducted by Saeed [33] showed a prevalence well beyond the range of the other studies, which could have contributed to the heterogeneity. However, after removing this study, the prevalence of oral candidiasis was found to be 3% (95% CI: 1—6%; heterogeneity: *I*^*2*^ = 0%, *P* = 0.39).

#### Petechiae

A meta-analysis of four studies yielded a prevalence rate of 8% (95% CI: 5—13%; heterogeneity: I2 = 37%, *P* = 0.19; 298 patients) for petechiae. (Supplementary Figure S15)

#### Cheilitis

A meta-analysis of three studies revealed that the prevalence of cheilitis was 6% (95% CI: 2—10%; I2 = 0%, *P* = 0.43; 150 patients). (Supplementary Figure S16)

#### Central erythema with white speckles or striae

In two studies [34, 35], the presence of central erythema with white speckles or striae in SLE patients was described. Khatibi et al. [34] reported a prevalence of 13.8%, while Mayssoun [35] reported a prevalence of 11.7%.

## Discussion

This is the first systematic review and meta-analysis to evaluate the prevalence of OMLs in SLE patients. Our study revealed that the estimated prevalence of OMLs was 31% in SLE patients, with the majority of cases (30%) manifesting as oral ulcers. Due to the high heterogeneity among the included studies, our findings should be interpreted with caution. The heterogeneity in this study can be attributed to geographic region, race, age of onset, disease status, and sample size.

We discovered a strong correlation between disease activity and OMLs in this study. This is comparable to the finding of Urman et al. [36] in which the prevalence of OMLs was associated with increased clinical disease activity. In addition, we found that the prevalence of OMLs was significantly different between active SLE (77%) and inactive SLE (18%). Nevertheless, since OMLs may be asymptomatic in patients with dormant SLE, our findings will need to be confirmed in a larger cohort to avoid underestimation.

The relationship between the prevalence of OMLs and gender in SLE patients has been controversial. Wang et al. [37] reported that the prevalence of oral ulcers was significantly lower in female patients than in male patients due to the influence of sex hormones, which is supported by the findings of numerous other studies [38, 39]. However, our analysis did not find any significant difference in the prevalence of OMLs between male (34%) and female (37%) SLE patients, which is in line with the conclusions of Murphy et al. [40] Therefore, further research of the association between OML prevalence and gender in SLE patients is warranted.

It is important to understand the clinical oral symptoms of systemic diseases such as SLE, since oral lesions are a common manifestation of systemic disorders and are frequently the only or defining marker of disease. The prompt diagnosis of these disorders by primary care physicians can offer patients with early access to specialized care before the disease progresses and causes misery, loss of productivity, and diminished quality of life. According to the results of our subgroup analysis by year of publication, the reported prevalence of OMLs among SLE patients did not change significantly across different year groups. Though, it is worth noting that 54 of the 109 included papers were published after 2016, indicating that more researchers are now focused on the prevalence of OMLs in SLE patients. Furthermore, our results showed that the prevalence of OMLs was significantly higher in studies with a sample size below 100, suggesting that smaller studies may overestimate the prevalence of OMLs in SLE patients.

Regional variations in the prevalence of OML and oral ulcers among SLE patients were evident in our subgroup analysis, possibly attributable to genetic, environmental, or cultural factors, as well as disparities in healthcare access and utilization. We found that the prevalence of oral ulcers in SLE patients was generally higher in tropical countries than in non-tropical countries, and we speculate that this difference in prevalence may be due to several factors. First, genetic factors play an important role in the pathogenesis of SLE and involvement of different organs and tissues [41]. It has been demonstrated that the prevalence of oral mucositis varies among SLE patients of different races and ethnic groups [42], which is consistent with our results. Our study revealed higher prevalence of oral ulcers in Indians and Malays compared to whites and blacks with SLE. This difference may be attributed to genetic differences among ethnic groups or even differences in living habits among regions. However, since only three of the included studies involved Indians [43, 44] or Malays [43, 45], further research is needed to determine the correlation between ethnic origin and oral ulcers in SLE patients. Second, environmental variables may contribute to SLE and accelerate its progression. The activity and prevalence of SLE have been reported to be associated with UV exposure, temperature, atmospheric pressure, mean humidity, wind speed, and precipitation [46]. Among them, UV radiation appears to play a large role in the development of oral ulcers. The study by Bijl et al. [47] has revealed that UV exposure can lead to the accumulation of apoptotic cells, which have been considered to be the major source of autoantigens in SLE [48]. Genetically susceptible individuals may produce pathogenic antibodies that recognize self-antigens and form antigen-antibody complexes, thereby inducing type III hypersensitivity. This in turn induces keratinocyte degeneration in the basement membrane of the oral mucosa, resulting in pathological changes in the oral mucosa [49]. Our study indicates that regions with a tropical climate and abundant solar radiation, such as South Asia, Southeast Asia, South America, Africa, and West Asia, are associated with a heightened risk for oral ulcers. These findings are consistent with Wright CY et al.‘s [50] report of high UV exposure in South Africa, where the prevalence of oral ulcers among SLE patients is as high as 50%. Third, differences in the number of studies conducted in various geographical regions may have resulted in data heterogeneity. Extensive research has been conducted in Europe, with a sample size of 16,950 subjects, in contrast to only one study in Oceania involving 24 subjects. Finally, other socioeconomic factors may also affect certain ethnic groups, such as access to health services and adherence to treatment, which may significantly affect disease progression.

While SLE predominantly affects women of reproductive age, it can also affect children and the elderly. It has been reported that the age of onset may have a significant impact on clinical presentation, morbidity, mortality, and treatment response of SLE patients [51]. Our subgroup analysis revealed that the prevalence of oral ulcer was higher in patients with childhood-onset SLE (41%) than in patients with adult-onset (26%) and late-onset SLE (26%). This is similar to previous studies that reported an 11.4-37% prevalence of oral ulcers in juvenile SLE, which is significantly higher than that in adult SLE [52, 53]. This difference is most likely due to the more severe disease in the adolescent population and a milder course in adults [53, 54].

The prevalence of other oral mucosal diseases appears to be relatively low in patients with SLE, with all rates seemingly below 10%. Specifically, white plaque has a prevalence of 3%, cheilitis 6%, petechiae 8%, and oral candidiasis and erythema both have a prevalence of 9%. After excluding studies with high heterogeneity, the prevalence of candidiasis was found to be 3% and the rate of erythema was found to be 7%. However, due to the limited number of studies included in the meta-analysis, further research is still required.

There are several limitations to this study. Although the studies included in this review provided diverse samples from 6 continents, it may be inaccurate to assume that the prevalence of oral involvement in SLE patients observed here can be extrapolated worldwide. Access to dentists and dermatologists who normally diagnose and treat SLE patients is limited in certain regions of the world, which impedes the collection of exhaustive epidemiological data. Since most studies only discussed the prevalence of oral ulcers, it would be misleading to assume that the data represent the prevalence of all oral mucosal disorders. Moreover, several of the investigations were cross-sectional. Oral signs may not have been apparent at the time of data collection due to the variation in SLE severity at the time of onset and the huge heterogeneity in treatment response. Therefore, longitudinal investigations are important to offer more insight into the prevalence of oral symptoms of SLE across different disease stages.

## Conclusion

Current evidence suggests that there is a 31% overall prevalence of oral mucosal involvement in SLE patients. Despite the significant heterogeneity among studies, individuals of Indian, Malay, and Caucasian descent who reside in tropical areas and experience childhood illnesses have a higher likelihood of developing oral ulcers. Given that nearly one-third of SLE patients exhibit OMLs, physicians need to be familiarized with the SLE-related signs for early diagnosis and proper management of patients.

### Electronic supplementary material

Below is the link to the electronic supplementary material.

Supplementary Material 1

## Data Availability

The datasets used and/or analyzed during the current study are available from the corresponding author on reasonable request.

## List of abbreviations

OMLs: Oral mucosal lesions
SLE: Systemic lupus erythematosus
NOS: Newcastle-Ottawa Scale
AHRQ: Agency for Healthcare Research and Quality
ACR: The American College of Rheumatology
SLICC: Systemic Lupus International Collaborating Clinics
