# Supplementary material for: Prevalence of oral mucosal lesions in patients with systemic Lupus Erythematosus: a systematic review and meta-analysis
Source: BMC Oral Health. 2023 Dec 21;23:1030. doi: 10.1186/s12903-023-03783-5 (PMC10734171; doi:10.1186/s12903-023-03783-5)
Supplement: Supplementary file 1 — Supplementary Material 1 [file 12903_2023_3783_MOESM1_ESM.doc]

**BMC Oral Health**

**Prevalence of oral mucosal lesions in patients with systemic lupus erythematosus: A systematic review and meta-analysis**

**Supplementary material**

*Supplementary search, figures, tables and references*

Fei Du, Wanying Qian, Xinna Zhang, Le Zhang, Jianwei Shang*

**Corresponding author:**

*Jianwei Shang, MD

Department of Oral Pathology, Tianjin Stomatological Hospital, Hospital of Stomatology, Nankai University, No. 75 Dagu North Rd, Tianjin, 300041, China.

E-mail: luckyedu123@126.com.

**Table of contents**

1.Search strategy (Supplementary Table S1)  **3**

2.Characteristics of analyzed studies. (Supplementary Table S2)  **7**

3.Subgroup analysis of OMLs prevalence among SLE patients. (Supplementary Table S3)  **15**

4.Subgroup analysis of oral ulcer prevalence among SLE patients. (Supplementary Table S4)  **16**

5.Prevalence of oral ulcers among SLE patients. (Forest plot of the included studies).(Supplementary Figure S1)  **17**

6.Oral mucosal diseases prevalence among SLE patients by sex. (Supplementary Figure S2)  **18**

7.Oral mucosal diseases prevalence among SLE patients by year of publication. (Supplementary Figure S3)  **19**

8.Oral mucosal diseases prevalence among SLE patients by region. (Supplementary Figure S4)  **20**

9.Oral mucosal diseases prevalence among SLE patients by disease status. (Supplementary Figure S5)  **21**

10.Oral mucosal diseases prevalence among SLE patients by sample size. (Supplementary Figure S6)  **22**

11.Sensitivity Analysis（Supplementary Figure S7）  **23**

12.Publication Bias（Supplementary Figure S8）  **24**

13.Oral ulcers prevalence among SLE patients by age of onset. (Supplementary Figure S9)  **25**

14.Oral ulcers prevalence among SLE patients by race. (Supplementary Figure S10)  **26**

15.Oral ulcers prevalence among SLE patients by region. (Supplementary Figure S11)  **27**

16.Prevalence of erythema among SLE patients (Forest plot of the included studies). (Supplementary Figure S12)  **28**

17.Prevalence of white plaque among SLE patients (Forest plot of the included studies). (Supplementary Figure S13)  **29**

18.Prevalence of oral candidiasis among SLE patients (Forest plot of the included studies). (Supplementary Figure S14)  **30**

19.Prevalence of petechiae among SLE patients (Forest plot of the included studies). (Supplementary Figure S15)  **31**

20.Prevalence of cheilitis among SLE patients (Forest plot of the included studies). (Supplementary Figure S16)  **32**

21.Supplementary references (113 studies included in the meta-analysis)  **33**

22.PRISMA 2020 Checklist  **41**

1. **S****earch strategy (Supplementary Table S1)**

**Table S1.** Search strategy for each database, number of results, and execution date.

| **Database** | **Query** | **Results** | **Upper date limit** |
| --- | --- | --- | --- |
| PubMed | ("lupus erythematosus, systemic"[MeSH Terms] OR ("lupus erythematosus systemic"[Title/Abstract] OR "systemic lupus erythematosus"[Title/Abstract] OR "lupus erythematosus disseminatus"[Title/Abstract] OR "libman sacks disease"[Title/Abstract] OR "disease libman sacks"[Title/Abstract] OR "libman sacks disease"[Title/Abstract])) AND ("Mouth Mucosa"[MeSH Terms] OR ("Mouth Mucosa"[Title/Abstract] OR "mucosa mouth"[Title/Abstract] OR "oral mucosa"[Title/Abstract] OR "mucosa oral"[Title/Abstract] OR "buccal mucosa"[Title/Abstract] OR "oral mucosal"[Title/Abstract]) OR ("Oral Manifestations"[MeSH Terms] OR ("Oral Manifestations"[Title/Abstract] OR "manifestation oral"[Title/Abstract] OR "manifestations oral"[Title/Abstract] OR "oral manifestation"[Title/Abstract])) OR ("pathology, oral"[MeSH Terms] OR ("pathology oral"[Title/Abstract] OR ("Oral"[Title/Abstract] AND "maxillofacial pathology"[Title/Abstract]) OR "oral pathology"[Title/Abstract] OR "pathology maxillofacial"[Title/Abstract] OR "maxillofacial pathology"[Title/Abstract])) OR ("Mouth Diseases"[MeSH Terms] OR ("Mouth Diseases"[Title/Abstract] OR "disease mouth"[Title/Abstract] OR "diseases mouth"[Title/Abstract] OR "mouth disease"[Title/Abstract])) OR ("Mouth"[MeSH Terms] OR ("Mouth"[Title/Abstract] OR "oral cavity"[Title/Abstract] OR "cavity oral"[Title/Abstract] OR "cavitas oris"[Title/Abstract] OR "vestibule of the mouth"[Title/Abstract] OR (("vestibule, labyrinth"[MeSH Terms] OR ("Vestibule"[All Fields] AND "labyrinth"[All Fields]) OR "labyrinth vestibule"[All Fields] OR "Vestibule"[All Fields] OR "vestibules"[All Fields]) AND "Oris"[Title/Abstract]) OR "oral cavity proper"[Title/Abstract] OR (("Mouth"[MeSH Terms] OR "Mouth"[All Fields] OR "mouths"[All Fields] OR "mouth s"[All Fields] OR "mouthed"[All Fields] OR "mouthful"[All Fields] OR "mouthfuls"[All Fields] OR "mouthing"[All Fields]) AND "cavity proper"[Title/Abstract]) OR "cavitas oris propria"[Title/Abstract])) OR ("oral lesion"[Title/Abstract] OR "oral lesions"[Title/Abstract] OR "oral alteration"[Title/Abstract] OR "oral alterations"[Title/Abstract] OR "oral pathologies"[Title/Abstract] OR "oral complications"[Title/Abstract] OR "oral complication"[Title/Abstract] OR "oral changes"[Title/Abstract] OR "oral change"[Title/Abstract] OR "oral diseases"[Title/Abstract] OR "oral disease"[Title/Abstract] OR "aphtae"[Title/Abstract] OR "aphtous"[Title/Abstract] OR "burning mouth"[Title/Abstract] OR "candidiasis oral"[Title/Abstract] OR "oral candidiasis"[Title/Abstract] OR "leukoedema oral"[Title/Abstract] OR "oral leukoedema"[Title/Abstract] OR "lichen planus oral"[Title/Abstract] OR "oral lichen planus"[Title/Abstract] OR "cheilitis"[Title/Abstract] OR "herpes labialis"[Title/Abstract] OR "labial herpes"[Title/Abstract] OR "oral herpes"[Title/Abstract] OR "oral mucositis"[Title/Abstract] OR "oral ulcer"[Title/Abstract] OR "oral ulcer"[Title/Abstract] OR "mouth ulcer"[Title/Abstract] OR "Stomatitis"[Title/Abstract] OR "stomatitis aphthous"[Title/Abstract] OR "stomatitis herpetic"[Title/Abstract] OR "glossitis benign migratory"[Title/Abstract] OR "geographic tongue"[Title/Abstract] OR "gingival epulis"[Title/Abstract] OR "oral papilloma"[Title/Abstract] OR "pyogenic granuloma"[Title/Abstract] OR "pemphigoid bullous"[Title/Abstract] OR "Pemphigoid"[Title/Abstract] OR "Pemphigoids"[Title/Abstract] OR "Pemphigus"[Title/Abstract] OR "Buccal"[Title/Abstract])) AND ("Prevalence"[MeSH Terms] OR ("Prevalences"[Title/Abstract] OR "period prevalence"[Title/Abstract] OR "period prevalences"[Title/Abstract] OR "prevalence period"[Title/Abstract] OR "point prevalence"[Title/Abstract] OR "point prevalences"[Title/Abstract] OR "prevalence point"[Title/Abstract] OR "prevalence study"[Title/Abstract]) OR "Epidemiology"[MeSH Terms] OR ("social epidemiology"[Title/Abstract] OR (("Epidemiologies"[All Fields] OR "Epidemiology"[MeSH Subheading] OR "Epidemiology"[All Fields] OR "Epidemiology"[MeSH Terms] OR "epidemiology s"[All Fields]) AND "Social"[Title/Abstract]) OR "epidemiology social"[Title/Abstract] OR "social epidemiologies"[Title/Abstract] OR "Epidemiology"[Title/Abstract] OR "epidemiological"[Title/Abstract] OR "clinical epidemiology"[Title/Abstract] OR ((("confound"[All Fields] OR "confounded"[All Fields] OR "confounder"[All Fields] OR "confounders"[All Fields] OR "confounding"[All Fields] OR "confounds"[All Fields]) AND ("factor"[All Fields] OR "factor s"[All Fields] OR "factors"[All Fields])) AND "Epidemiology"[Title/Abstract]) OR "confounding factors epidemiologic"[Title/Abstract] OR "controlled before after studies"[Title/Abstract] OR ("controlled before"[Title/Abstract] AND "after studies"[Title/Abstract]) OR ("controlled before"[Title/Abstract] AND "after study"[Title/Abstract]) OR "controlled before after studies"[Title/Abstract] OR "effect modifier epidemiologic"[Title/Abstract] OR ((("effect"[All Fields] OR "effecting"[All Fields] OR "effective"[All Fields] OR "effectively"[All Fields] OR "effectiveness"[All Fields] OR "effectivenesses"[All Fields] OR "effectives"[All Fields] OR "effectivities"[All Fields] OR "effectivity"[All Fields] OR "effects"[All Fields]) AND ("modifiable"[All Fields] OR "modified"[All Fields] OR "modifier"[All Fields] OR "modifiers"[All Fields] OR "modifies"[All Fields] OR "modify"[All Fields] OR "modifying"[All Fields])) AND "Epidemiology"[Title/Abstract]) OR ((("effect"[All Fields] OR "effecting"[All Fields] OR "effective"[All Fields] OR "effectively"[All Fields] OR "effectiveness"[All Fields] OR "effectivenesses"[All Fields] OR "effectives"[All Fields] OR "effectivities"[All Fields] OR "effectivity"[All Fields] OR "effects"[All Fields]) AND ("modifiable"[All Fields] OR "modified"[All Fields] OR "modifier"[All Fields] OR "modifiers"[All Fields] OR "modifies"[All Fields] OR "modify"[All Fields] OR "modifying"[All Fields])) AND "psychology"[Title/Abstract]) OR "environmental epidemiology"[Title/Abstract] OR "epidemiologic confounding factors"[Title/Abstract] OR "epidemiologic effect modifier"[Title/Abstract] OR "epidemiologic factors"[Title/Abstract] OR "epidemiologic methods"[Title/Abstract] OR "epidemiologic research"[Title/Abstract] OR "epidemiologic research design"[Title/Abstract] OR "epidemiologic studies"[Title/Abstract] OR "epidemiologic study characteristics"[Title/Abstract] OR "epidemiologic study characteristics as topic"[Title/Abstract] OR "epidemiologic survey"[Title/Abstract] OR "epidemiological research"[Title/Abstract] OR "epidemiology model"[Title/Abstract] OR "epidemiometry"[Title/Abstract] OR "historically controlled study"[Title/Abstract] OR "interrupted time series analysis"[Title/Abstract] OR "precipitating factors"[Title/Abstract] OR "sampling studies"[Title/Abstract]) OR "Cross-Sectional Studies"[MeSH Terms] OR ("Cross-Sectional Studies"[Title/Abstract] OR "Cross-Sectional Studies"[Title/Abstract] OR "cross sectional study"[Title/Abstract] OR "studies cross sectional"[Title/Abstract] OR "study cross sectional"[Title/Abstract] OR "cross sectional analysis"[Title/Abstract] OR "analyses cross sectional"[Title/Abstract] OR "cross sectional analyses"[Title/Abstract] OR "disease frequency surveys"[Title/Abstract] OR "cross sectional survey"[Title/Abstract] OR "cross sectional survey"[Title/Abstract] OR "cross sectional surveys"[Title/Abstract] OR "survey cross sectional"[Title/Abstract] OR "surveys cross sectional"[Title/Abstract] OR (("survey s"[All Fields] OR "surveyed"[All Fields] OR "surveying"[All Fields] OR "surveys and questionnaires"[MeSH Terms] OR ("Surveys"[All Fields] AND "questionnaires"[All Fields]) OR "surveys and questionnaires"[All Fields] OR "Survey"[All Fields] OR "Surveys"[All Fields]) AND "disease frequency"[Title/Abstract]) OR (("Disease"[MeSH Terms] OR "Disease"[All Fields] OR "Diseases"[All Fields] OR "disease s"[All Fields] OR "diseased"[All Fields]) AND "frequency survey"[Title/Abstract]) OR (("survey s"[All Fields] OR "surveyed"[All Fields] OR "surveying"[All Fields] OR "surveys and questionnaires"[MeSH Terms] OR ("Surveys"[All Fields] AND "questionnaires"[All Fields]) OR "surveys and questionnaires"[All Fields] OR "Survey"[All Fields] OR "Surveys"[All Fields]) AND "disease frequency"[Title/Abstract]) OR "analysis cross sectional"[Title/Abstract] OR "analyses cross sectional"[Title/Abstract] OR "analysis cross sectional"[Title/Abstract] OR "cross sectional analyses"[Title/Abstract] OR "cross sectional analysis"[Title/Abstract] OR "prevalence studies"[Title/Abstract] OR "prevalence study"[Title/Abstract] OR "studies prevalence"[Title/Abstract] OR "study prevalence"[Title/Abstract] OR "cross sectional design"[Title/Abstract] OR "cross sectional research"[Title/Abstract])) | 200 | January  2022 |
| Embase | ((('oral mucosal disease'/exp OR ('mouth mucosa disease':ab,ti OR 'oral mucosa disease':ab,ti OR 'oral mucosa diseases':ab,ti OR 'oral mucosa disorder':ab,ti OR 'oral mucosa disorders':ab,ti OR 'oral mucosa lesion':ab,ti OR 'oral mucosa lesions':ab,ti OR 'oral mucosal conditions':ab,ti OR 'oral mucosal diseases':ab,ti OR 'oral mucosal disorder':ab,ti OR 'oral mucosal disorders':ab,ti OR 'oral mucosal lesion':ab,ti OR 'oral mucosal lesions':ab,ti OR 'oral mucosal pathology':ab,ti OR 'oral mucosal disease':ab,ti)) OR ('buccal mucosa'/exp OR ('buccal mucosae':ab,ti OR 'buccal mucous membrane':ab,ti OR 'cheek defect, mucosal surface':ab,ti OR 'cheek mucosa':ab,ti OR 'cheek mucosa defect':ab,ti OR 'cheek mucosae':ab,ti OR 'cheek pouch mucosa':ab,ti OR 'mucosal surface cheek defect':ab,ti OR 'palatal mucosa':ab,ti OR 'buccal mucosa':ab,ti)) OR ('dentistry'/exp OR ('dental medicine':ab,ti OR 'dental specialties':ab,ti OR 'dental specialty':ab,ti OR 'dental system':ab,ti OR 'occupational dentistry':ab,ti OR 'pathology, oral':ab,ti OR 'specialties, dental':ab,ti OR 'state dentistry':ab,ti OR 'dentistry':ab,ti)) OR ('mouth disease'/exp OR ('diagnosis, oral':ab,ti OR 'edentulous mouth':ab,ti OR 'leukoedema, oral':ab,ti OR 'leukooedema, oral':ab,ti OR 'mouth abnormalities':ab,ti OR 'mouth diseases':ab,ti OR 'mouth pathology':ab,ti OR 'mouth submucous fibrosis':ab,ti OR 'mouth, edentulous':ab,ti OR 'oral diagnosis':ab,ti OR 'oral disease':ab,ti OR 'oral leukoedema':ab,ti OR 'oral leukooedema':ab,ti OR 'oral manifestations':ab,ti OR 'oral pathology':ab,ti OR 'oral submucous fibrosis':ab,ti OR 'stomatognathic diseases':ab,ti OR 'mouth disease':ab,ti)) OR 'mouth'/exp OR ('oral lesion':ab,ti OR 'oral lesions':ab,ti OR 'oral alteration':ab,ti OR 'oral alterations':ab,ti OR 'oral pathologies':ab,ti OR 'oral complications':ab,ti OR 'oral complication':ab,ti OR 'oral changes':ab,ti OR 'oral change':ab,ti OR 'oral diseases':ab,ti OR 'oral disease':ab,ti OR 'aphtae':ab,ti OR 'aphtous':ab,ti OR 'burning mouth':ab,ti OR 'candidiasis, oral':ab,ti OR 'oral candidiasis':ab,ti OR 'lichen planus, oral':ab,ti OR 'oral lichen planus':ab,ti OR 'cheilitis':ab,ti OR 'herpes labialis':ab,ti OR 'labial herpes':ab,ti OR 'oral herpes':ab,ti OR 'oral mucositis':ab,ti OR 'oral ulcer':ab,ti OR 'mouth ulcer':ab,ti OR 'stomatitis':ab,ti OR 'stomatitis, aphthous':ab,ti OR 'stomatitis, herpetic':ab,ti OR 'glossitis, benign migratory':ab,ti OR 'geographic tongue':ab,ti OR 'gingival epulis':ab,ti OR 'oral papilloma':ab,ti OR 'pyogenic granuloma':ab,ti OR 'pemphigoid, bullous':ab,ti OR 'pemphigoid':ab,ti OR 'pemphigoids':ab,ti OR 'pemphigus':ab,ti)) AND (('systemic lupus erythematosus'/exp) OR ('systemic lupus erythematosus':ab,ti OR 'dermatovisceritism, malignant':ab,ti OR 'disseminated lupus':ab,ti OR 'disseminated lupus erythematodes':ab,ti OR 'disseminated lupus erythematosis':ab,ti OR 'disseminated lupus erythematosus':ab,ti OR 'erythematodes visceralis':ab,ti OR 'lupovisceritis':ab,ti OR 'lupus erythematodes disseminatus':ab,ti OR 'lupus erythematosus disseminatus':ab,ti OR 'lupus erythematosus visceralis':ab,ti OR 'lupus erythematosus, systemic':ab,ti OR 'osler libman sacks disease':ab,ti OR 'systemic lupus erythematodes':ab,ti OR 'systemic lupus erythematosis':ab,ti OR 'systemic lupus erythematous':ab,ti))) AND (('prevalence'/exp OR ('prevalence':ab,ti OR 'prevalences':ab,ti OR 'period prevalence':ab,ti OR 'period prevalences':ab,ti OR 'prevalence, period':ab,ti OR 'point prevalence':ab,ti OR 'point prevalences':ab,ti OR 'prevalence, point':ab,ti OR 'prevalence study':ab,ti)) OR ('epidemiology'/exp OR ('social epidemiology':ab,ti OR 'epidemiologies, social':ab,ti OR 'epidemiology, social':ab,ti OR 'social epidemiologies':ab,ti OR 'epidemiology':ab,ti OR 'epidemiological':ab,ti OR 'clinical epidemiology':ab,ti OR 'confounding factors (epidemiology)':ab,ti OR 'confounding factors, epidemiologic':ab,ti OR 'controlled before after studies':ab,ti OR 'controlled before and after studies':ab,ti OR 'controlled before and after study':ab,ti OR 'controlled before-after studies':ab,ti OR 'effect modifier, epidemiologic':ab,ti OR 'effect modifiers (epidemiology)':ab,ti OR 'effect modifiers (psychology)':ab,ti OR 'environmental epidemiology':ab,ti OR 'epidemiologic confounding factors':ab,ti OR 'epidemiologic effect modifier':ab,ti OR 'epidemiologic factors':ab,ti OR 'epidemiologic methods':ab,ti OR 'epidemiologic research':ab,ti OR 'epidemiologic research design':ab,ti OR 'epidemiologic studies':ab,ti OR 'epidemiologic study characteristics':ab,ti OR 'epidemiologic study characteristics as topic':ab,ti OR 'epidemiologic survey':ab,ti OR 'epidemiological research':ab,ti OR 'epidemiology model':ab,ti OR 'epidemiometry':ab,ti OR 'historically controlled study':ab,ti OR 'interrupted time series analysis':ab,ti OR 'precipitating factors':ab,ti OR 'sampling studies':ab,ti)) OR ('cross-sectional study'/exp OR ('cross-sectional studies':ab,ti OR 'cross sectional studies':ab,ti OR 'cross-sectional study':ab,ti OR 'studies, cross-sectional':ab,ti OR 'study, cross-sectional':ab,ti OR 'cross sectional analysis':ab,ti OR 'analyses, cross sectional':ab,ti OR 'cross sectional analyses':ab,ti OR 'disease frequency surveys':ab,ti OR 'cross-sectional survey':ab,ti OR 'cross sectional survey':ab,ti OR 'cross-sectional surveys':ab,ti OR 'survey, cross-sectional':ab,ti OR 'surveys, cross-sectional':ab,ti OR 'surveys, disease frequency':ab,ti OR 'disease frequency survey':ab,ti OR 'survey, disease frequency':ab,ti OR 'analysis, cross-sectional':ab,ti OR 'analyses, cross-sectional':ab,ti OR 'analysis, cross sectional':ab,ti OR 'cross-sectional analyses':ab,ti OR 'cross-sectional analysis':ab,ti OR 'prevalence studies':ab,ti OR 'prevalence study':ab,ti OR 'studies, prevalence':ab,ti OR 'study, prevalence':ab,ti OR 'cross-sectional design':ab,ti OR 'cross-sectional research':ab,ti))) | 1440 | January  2022 |
| Google Scholar | （"Lupus Erythematosus, Systemic" OR "Systemic Lupus Erythematosus" OR "Lupus Erythematosus Disseminatus" OR "Libman-Sacks Disease" OR "Disease, Libman-Sacks" OR "Libman Sacks Disease"） AND （“Mouth Mucosa” OR “oral manifestations” OR “Pathology, Oral” OR “Mouth Diseases” OR “Mouth” OR “oral mucosal disease” OR “buccal mucosa”OR “dentistry mouth disease” OR "oral lesion" OR "oral lesions" OR "oral alteration" OR "oral alterations" OR "oral pathologies" OR "oral complications" OR "oral complication" OR "oral changes" OR "oral change" OR "oral diseases" OR "oral disease" OR "aphtae" OR "aphtous" OR "burning mouth" OR "candidiasis oral" OR "oral candidiasis" OR "leukoedema oral" OR "oral leukoedema" OR "lichen planus oral" OR "oral lichen planus" OR "cheilitis" OR "herpes labialis" OR "labial herpes" OR "oral herpes" OR "oral mucositis" OR "oral ulcer" OR "oral ulcer" OR "mouth ulcer" OR "Stomatitis" OR "stomatitis aphthous" OR "stomatitis herpetic" OR "glossitis benign migratory" OR "geographic tongue" OR "gingival epulis" OR "oral papilloma" OR "pyogenic granuloma" OR "pemphigoid bullous" OR "Pemphigoid" OR "Pemphigoids" OR "Pemphigus" OR "buccal"） AND (“prevalence” OR “Epidemiology” OR “epidemiological” OR “Cross-Sectional” OR “Crosssectional”) | 1966 | January 2022 |
| Cochrane Library | (MeSH descriptor: [Lupus Erythematosus, Systemic] explode all trees OR ((lupus erythematosus systemic):ti,ab,kw OR (systemic lupus erythematosus):ti,ab,kw OR (lupus erythematosus disseminatus):ti,ab,kw OR (libman sacks disease):ti,ab,kw OR (disease libman sacks):ti,ab,kw) OR libman sacks disease) AND ((MeSH descriptor: [Mouth Mucosa] explode all trees OR ((mouth mucosa):ti,ab,kw OR (mucosa mouth):ti,ab,kw OR (oral mucosa):ti,ab,kw OR (mucosa oral):ti,ab,kw OR (buccal mucosa):ti,ab,kw) OR oral mucosal) OR (MeSH descriptor: [Mouth Mucosa] explode all trees OR ((oral manifestations):ti,ab,kw OR (Manifestation, Oral):ti,ab,kw OR (Manifestations, Oral):ti,ab,kw OR (Oral Manifestation):ti,ab,kw)) OR (MeSH descriptor: [Mouth Diseases] explode all trees OR ((Mouth Diseases):ti,ab,kw OR (Disease, Mouth):ti,ab,kw OR (Diseases, Mouth):ti,ab,kw OR (Mouth Disease):ti,ab,kw)) OR (MeSH descriptor: [Mouth] explode all trees OR ((Mouth):ti,ab,kw OR (Oral Cavity):ti,ab,kw OR (Cavity, Oral):ti,ab,kw OR (Cavitas Oris):ti,ab,kw OR (Vestibule of the Mouth):ti,ab,kw) OR ((Vestibule Oris):ti,ab,kw OR (Oral Cavity Proper):ti,ab,kw OR (Mouth Cavity Proper):ti,ab,kw OR (Cavitas oris propria):ti,ab,kw)) OR ((oral lesion):ti,ab,kw OR (oral lesions):ti,ab,kw OR (oral alteration):ti,ab,kw OR (oral alterations):ti,ab,kw OR (oral pathologies):ti,ab,kw OR ((oral complications):ti,ab,kw OR (oral complication):ti,ab,kw OR (oral changes):ti,ab,kw OR (oral change):ti,ab,kw OR (oral diseases):ti,ab,kw) OR ((oral disease):ti,ab,kw OR (aphtae):ti,ab,kw OR (aphtous):ti,ab,kw OR (burning mouth):ti,ab,kw OR (Candidiasis, Oral):ti,ab,kw) OR ((oral Candidiasis):ti,ab,kw OR (Leukoedema, Oral):ti,ab,kw OR (oral Leukoedema):ti,ab,kw OR (Lichen Planus, Oral):ti,ab,kw OR (Oral Lichen Planus):ti,ab,kw) OR ((cheilitis):ti,ab,kw OR (herpes labialis):ti,ab,kw OR (labial herpes):ti,ab,kw OR (oral herpes):ti,ab,kw OR (oral mucositis):ti,ab,kw) OR ((Oral Ulcer):ti,ab,kw OR (oral ulcer):ti,ab,kw OR (mouth ulcer):ti,ab,kw OR (Stomatitis):ti,ab,kw OR (Stomatitis, Aphthous):ti,ab,kw) OR ((Stomatitis, Herpetic):ti,ab,kw OR (Glossitis, Benign Migratory):ti,ab,kw OR (Geographic Tongue):ti,ab,kw OR (gingival epulis):ti,ab,kw OR (oral papilloma):ti,ab,kw) OR ((pyogenic granuloma):ti,ab,kw OR (Pemphigoid, Bullous):ti,ab,kw OR (Pemphigoid):ti,ab,kw OR (Pemphigoids):ti,ab,kw OR (Pemphigus):ti,ab,kw) OR((buccal):ti,ab,kw))) AND ((MeSH descriptor: [Prevalence] explode all trees OR ((Prevalences):ti,ab,kw OR (Period Prevalence):ti,ab,kw OR (Period Prevalences):ti,ab,kw OR (Prevalence, Period):ti,ab,kw OR (Point Prevalence):ti,ab,kw) OR ((Point Prevalences):ti,ab,kw OR (Prevalence, Point):ti,ab,kw OR (prevalence study):ti,ab,kw)) OR (MeSH descriptor: [Epidemiology] explode all trees OR ((Social Epidemiology):ti,ab,kw OR (Epidemiologies, Social):ti,ab,kw OR (Epidemiology, Social):ti,ab,kw OR (Social Epidemiologies):ti,ab,kw OR (epidemiology):ti,ab,kw) OR ((epidemiological):ti,ab,kw OR (clinical epidemiology):ti,ab,kw OR (confounding factors (epidemiology)):ti,ab,kw OR (confounding factors, epidemiologic):ti,ab,kw OR (controlled before after studies):ti,ab,kw) OR ((controlled before and after studies):ti,ab,kw OR (controlled before and after study):ti,ab,kw OR (controlled before-after studies):ti,ab,kw OR (effect modifier, epidemiologic):ti,ab,kw OR (effect modifiers (epidemiology)):ti,ab,kw) OR ((effect modifiers (psychology)):ti,ab,kw OR (environmental epidemiology):ti,ab,kw OR (epidemiologic confounding factors):ti,ab,kw OR (epidemiologic effect modifier):ti,ab,kw OR (epidemiologic factors):ti,ab,kw) OR ((epidemiologic methods):ti,ab,kw OR (epidemiologic research):ti,ab,kw OR (epidemiologic research design):ti,ab,kw OR (epidemiologic studies):ti,ab,kw OR (epidemiologic study characteristics):ti,ab,kw) OR ((epidemiologic study characteristics as topic):ti,ab,kw OR (epidemiologic survey):ti,ab,kw OR (epidemiological research):ti,ab,kw OR (epidemiology model):ti,ab,kw OR (epidemiometry):ti,ab,kw) OR ((historically controlled study):ti,ab,kw OR (interrupted time series analysis):ti,ab,kw OR (precipitating factors):ti,ab,kw OR (sampling studies):ti,ab,kw)) OR (MeSH descriptor: [Cross-Sectional Studies] explode all trees OR ((Cross-Sectional Studies):ti,ab,kw OR (Cross Sectional Studies):ti,ab,kw OR (Cross-Sectional Study):ti,ab,kw OR (Studies, Cross-Sectional):ti,ab,kw OR (Study, Cross-Sectional):ti,ab,kw) OR ((Cross Sectional Analysis):ti,ab,kw OR (Analyses, Cross Sectional):ti,ab,kw OR (Cross Sectional Analyses):ti,ab,kw OR (Disease Frequency Surveys):ti,ab,kw OR (Cross-Sectional Survey):ti,ab,kw) OR ((Cross Sectional Survey):ti,ab,kw OR (Cross-Sectional Surveys):ti,ab,kw OR (Survey, Cross-Sectional):ti,ab,kw OR (Surveys, Cross-Sectional):ti,ab,kw OR (Surveys, Disease Frequency):ti,ab,kw) OR ((Disease Frequency Survey):ti,ab,kw OR (Survey, Disease Frequency):ti,ab,kw OR (Analysis, Cross-Sectional):ti,ab,kw OR (Analyses, Cross-Sectional):ti,ab,kw OR (Analysis, Cross Sectional):ti,ab,kw) OR ((Cross-Sectional Analyses):ti,ab,kw OR (Cross-Sectional Analysis):ti,ab,kw OR (Prevalence Studies):ti,ab,kw OR (Prevalence Study):ti,ab,kw OR (Studies, Prevalence):ti,ab,kw) OR ((Study, Prevalence):ti,ab,kw OR (cross-sectional design):ti,ab,kw OR (cross-sectional research):ti,ab,kw))) | 12 | January 2022 |
| Web of Science | TS=("systemic lupus erythematosus" OR "SLE" OR "lupus erythematosus, systemic") AND TS=("oral manifestation*" OR "mouth mucosa" OR "oral cavity" OR "oral lesion*" OR "oral ulcers" OR "oral disease*" OR "oral pathology" OR "oral disorder*") AND TS=("prevalence" OR "incidence" OR "epidemiology" OR "occurrence" OR "frequency" OR "distribution") | 121 | January 2022 |
| Total | 3739 | | |

1. **Characteristics of analyzed studies. (Supplementary Table S2)**

**Table S2**. Studies included assessing oral mucosal lesions prevalence in patients with SLE.

| **（author, year）country** | **region** | **study type** | **total** | **age(years)** | **overall prevalence of findings n (%)** | | | | | **partial prevalence of findings n (%)** | | | **Diagnostic criteria** | **AHRQ** | **AHRQ** | **NOS** | **NOS** |
| --- | --- | --- | --- | --- | --- | --- | --- | --- | --- | --- | --- | --- | --- | --- | --- | --- | --- |
| (Aurlene et al., 2020) India[1] | South Asia | cross-sectional survey | 500 | (31.7， NA)* | 164/500（32.8%） | active SLE | inactive SLE | male | female |  | active SLE | inactive SLE | ACR, 2012 | 7 | M |  |  |
|  |  |  |  |  |  | 138/176  (78.4%) | 26/324  (8%) | NA | NA | Aphthous ulcers | 90(18%) | 24(4.8%) |  |  |  |  |  |
|  |  |  |  |  |  |  |  |  |  |  |  |  |  |  |  |  |  |
|  |  |  |  |  |  |  |  |  |  | pigmented macules | 6(1.2%) | 0 |  |  |  |  |  |
|  |  |  |  |  |  |  |  |  |  | Papules | 6(1.2%) | 1(0.2%) |  |  |  |  |  |
|  |  |  |  |  |  |  |  |  |  | Aphthous ulcers with others | 36(7.2%) | 1(0.2%) |  |  |  |  |  |
| (Khatibi et al., 2012) Iran[2] | West Asia | cross-sectional survey | 188 | NA | 102(54.3%) | 73/99(73.7%) | 29/89(32.6%) | 12/26(46.2%) | 90/162(55.6%) | Oral ulcer | 53(28.1%) | | ACR | 7 | M |  |  |
|  |  |  |  |  |  |  |  |  |  | central erythema with white speckles or striae | 26(13.8%) | |  |  |  |  |  |
|  |  |  |  |  |  |  |  |  |  | erythema | 18(9.5%) | |  |  |  |  |  |
|  |  |  |  |  |  |  |  |  |  | white plaque | 5(2.6%) | |  |  |  |  |  |
| (Koh et al., 2020) South Korea[3] | East Asia | retrospective cohort study | 413 | (30.9，12.9)* | 145（35.1%） | NA | NA | NA | NA | Oral ulcer | 145(35.1%) | | revised ACR,1997 |  |  | 8 | H |
| (Li et al., 2017) China[4] | East Asia | retrospective cohort study | 552 | (35.3,14.5) * | 94(17.0%) | NA | NA | NA | NA | Oral ulcer | 94(17.0%) | | revised ACR,1997 |  |  | 5 | M |
| (Zhao et al., 2013) China[5] | East Asia | retrospective cohort study | 113 | (26.0，3.2)* | 12(10.6%) | NA | NA | NA | NA | Oral ulcer | 12/113(10.6%) | | revised ACR,1997 |  |  | 5 | M |
|  |  |  |  |  |  |  |  |  |  |  |  |  |  |  |  |  |  |
| (Abd El Monem Teama et al., 2021) Egypt[6] | Africa | cross-sectional study | 100 | (12.91,1.94) * | 33(33.0%) | NA | NA | NA | NA | Oral ulcer | 33/100（33.0%） | | Systemic Lupus International Collaborating Clinics classification criteria | 6 | M |  |  |
| (Meyer et al., 2020) German and French[7] | Europe | cross-sectional study | 990 | (43.5,16.8) * | 261/990(26.4%) | NA | NA | NA | NA | Oral ulcer | 261/990(26.4%) | | revised ACR,1997 | 4 | M |  |  |
| (Mao et al., 2020) China[8] | East Asia | retrospective cohort study | 3140 | median (range):37(26-47) | 244(7.8%) | NA | NA | NA | NA | Oral ulcer | 244(7.8%) | | ACR |  |  | 6 | M |
| (Alonso et al., 2014) Spain[9] | Europe | cross-sectional study | 150 | （44.5，19.2）* | 22（14.7%） | NA | NA | 2/23(8.7%) | 20/127(15.7%) | Oral ulcer | 22(14.7%) | | ACR, dermatologists, or internal  medicine staff members | 8 | H |  |  |
| (To et al., 2009) China[10] | East Asia | prospective cohort study | 1082 | (30.5,12.7) * | 129/1082(12.0%) | NA | NA | NA | NA | Oral ulcer | 129(12.9%) | | ACR |  |  | 7 | M |
| (Wang & Gao, 2021) China[11] | East Asia | cross-sectional study | 105 | (41.36,5.28) * | 27(25.71%) | NA | NA | 10/21(47.6%) | 17/84(20.2%) | Oral ulcer | 27(25.71%) | | ACR | 7 | M |  |  |
| (Tejera Segura et al., 2021) Spain[12] | Europe | cross-sectional study | 3464 | (35.2,14.7) * | 1603(46.3%) | NA | NA | NA | NA | Oral ulcer | 1603(46.3%) | | ACR | 5 | M |  |  |
| (Soltani et al., 2021) Iran[13] | West Asia | cross-sectional study | 130 | (31.5,8.3) * | 15(11.5%) | NA | NA | NA | NA | Recurrent oral aphthosis | 15(11.5%) | | ACR | 7 | M |  |  |
|  |  |  |  |  |  |  |  |  |  |  |  |  |  |  |  |  |  |
| (Sieiro Santos et al., 2021) Spain[14] | Europe | case-control study | 403 | (55.4,8.2) * | 30(7.4%) | NA | NA | NA | NA | Oral ulcer | 30/403(7.4%) | | SLICC |  |  | 7 | M |
|  |  |  |  |  |  |  |  |  |  |  |  |  |  |  |  |  |  |
| (Lu et al., 2021) China[15] | East Asia | retrospective, cross-sectional study | 125 | median (range):36.0 (13.0–81.0) | 7(5.6%) | NA | NA | NA | NA | Oral ulcer | 7(5.6%) | | revised ACR,1997 | 7 | M |  |  |
| (Costa Pires et al., 2021) Portugal[16] | Europe | Single Centre Cohort Analysis | 707 | (28.99,13.32) * | 185(26.2%) | NA | NA | NA | NA | Oral ulcer | 185/707(26.2%) | | NA |  |  | 7 | M |
|  |  |  |  |  |  |  |  |  |  |  |  |  |  |  |  |  |  |
| (Quevedo Mayorga et al., 2022) Colombia[17] | South America | Retrospective cross-sectional study | 122 | (39,1.5) * | 47(38.5%) | NA | NA | NA | NA | Oral ulcer | 47/122(38.5%) | | SLICC, 2012 | 6 | M |  |  |
|  |  |  |  |  |  |  |  |  |  |  |  |  |  |  |  |  |  |
| (Othmani et al., 2002) Tunisia[18] | Africa | retrospective cohort study | 295 | 31.75(10-63) * | 46(15.7%) | NA | NA | 3/24(12.5%) | 43/271(16%) | Oropharyngeal ulcer | 46(15.7%) | | ACR |  |  | 6 | M |
|  |  |  |  |  |  |  |  |  |  |  |  |  |  |  |  |  |  |
| (Mok et al., 2005) Hong Kong (China) and Baltimor[19] | East Asia | prospective cohort study | 625 | (35.7，14)* | 226(36.2%) | NA | NA | NA | NA | Oral ulcers (East Asian) | 43/258(17%) | | ACR |  |  | 8 | H |
|  |  |  |  |  |  |  |  |  |  | Oral ulcers (Black race) | 52/140(37%) | |  |  |  |  |  |
|  |  |  |  |  |  |  |  |  |  | Oral ulcers (Caucasians) | 131/227(58%) | |  |  |  |  |  |
| (Wang et al., 2007) China[20] | East Asia | retrospective cohort study | 695 | (30.2,10.5) * | 113(16.3%) | NA | NA | NA | NA | Oral ulcer | 113/695(16.3%) | | ACR,1997 |  |  | 8 | H |
| (AlSaleh et al., 2008) Dubai[21] | West Asia | retrospective cohort study | 151 | (28.9,0.8) * | 46(30.5%) | NA | NA | NA | NA | Oral ulcer | 46/151(30.5%) | | ACR,1997 |  |  | 7 | M |
| (Mongkoltanatus et al., 2008) Thai[22] | Southeast Asia | retrospective cohort study | 111 | (34.5,12.4) * | 34(30.6%) | NA | NA | 12/37(32.4%) | 22/74(29.7%) | Oral ulcer | 34/111(30.6%) | | ACR |  |  | 7 | M |
|  |  |  |  |  |  |  |  |  |  |  |  |  |  |  |  |  |  |
| (Nazarinia et al., 2008) Iran[23] | West Asia | prospective cohort study | 410 | 30.27(3-78) * | 114(27.8%) | NA | NA | NA | NA | Oral ulcer | 114/410(27.8%) | | ACR,1997 |  |  | 7 | M |
| (Ramírez Gómez et al., 2008) Latin America[24] | South America | multicenter, multinational, prospective inception cohort | 1214 | Children-onset (IQR)(<18years)：15.3 (13.2–16.7)*years (n=230)；  Adult-onset SLE (IQR)(≧18years)：29.1 (23.2–37.5) * years(n=984) | 506（41.7%） | NA | NA | NA | NA | Oral ulcers (Children-onset) | 113/230(49.1%) | | diagnosis of SLE by a qualified internist  or rheumatologist ACR not mandatory |  |  | 7 | M |
|  |  |  |  |  |  |  |  |  |  | Oral ulcers (Adult-onset) | 393/984(39.9%) | |  |  |  |  |  |
| (Al Arfaj & Khalil, 2009) Saudi Arabia[25] | West Asia | retrospective cohort study | 624 | (25.3,11.4) * | 244(39.1%) | NA | NA | NA | NA | Oral ulcer | 244(39.1%) | | ACR |  |  | 8 | H |
| (Kole & Ghosh, 2009) India[26] | South Asia | retrospective cohort study | 150 | (30, NA) * | 85(56.67%) | NA | NA | NA | NA | Oral ulcer | 85/150(56.67%) | | ACR,1982 |  |  | 7 | M |
| (Mantovani et al., 2010) Brazil[27] | South America | retrospective cohort study | 141 | (36.2,11.4) * | 71(50.4%) | NA | NA | NA | NA | Oral ulcer | 71/141(50.4%) | | ACR |  |  | 6 | M |
| (Umbelino Júnior et al., 2010) Brazil[28] | South America | cross-sectional study | 155 | (41.2,12.3) * | 10(6.5%) | NA | NA | NA | NA | Oral candidiasis | 6/155(4%) | | ACR | 6 | M |  |  |
| (Alonso-Perez et al., 2011) Spain[29] | Europe | cohort study | 1413 | (31.1,13.1) * | 396(28.0%) | NA | NA | NA | NA | Oral ulcer | 396/1413(28.0%) | | ACR |  |  | 5 | M |
| (Jönsen et al., 2011) Sweden[30] | Europe | prospective cohort study | 669 | NA | 218(32.6%) | NA | NA | NA | NA | Oral ulcer | 218/669(32.6%) | | ACR |  |  | 6 | M |
| (Stefanidou et al., 2011) Greece[31] | Europe | retro-prospective epidemiological study | 594 | NA | 94/594(15.8%) | NA | NA | 7/59(11.9%) | 87/535(16.3) | Oral ulcer | 94/594(15.8%) | | the 11 revised ACR criteria |  |  | 6 | M |
| (Weckerle et al., 2011) America[32] | North America | cross-sectional study | 1089 | NA | 365（33.5%） | NA | NA | NA | NA | Oral ulcer (Black race) | 103/387(27%) | | ACR | 5 | M |  |  |
|  |  |  |  |  |  |  |  |  |  | Oral ulcer (Caucasians) | 262/702(37%) | |  |  |  |  |  |
| (Pettersson et al., 2012) Sweden[33] | Europe | cross-sectional study | 324 | median (range): (IQR)48 (35–58) | 110(34%) | NA | NA | NA | NA | Oral ulcer | 110/324(34%) | | ACR,1982 | 8 | H |  |  |
| (Sánchez et al., 2012) America[34] | North America | cohort study | 5645 | NA | 2534（44.9%） | NA | NA | NA | NA | Oral ulcer | 2534/5645(44.9%) | | ACR |  |  | 5 | M |
| (Lee et al., 2013) China[35] | East Asia | retrospective cohort study | 189 | (12.62,2.77) * | 66(34.92%) | NA | NA | 6/25(24%) | 60/164(36.59%) | Oral ulcer | 66/189(34.92%) | | ACR |  |  | 8 | H |
| (Li et al., 2013) China[36] | East Asia | cross-sectional retrospective study | 1928 | (29.8,13) * | 376(19.5%) | NA | NA | NA | NA | Oral ulcer | 376/1928919.5%） | | ACR | 6 | M |  |  |
| (Fredi et al., 2014) Italy[37] | Europe | retrospective cohort study | 540 | (34.01,12.8) * | 178(33.0%) | NA | NA | NA | NA | Oral ulcer | 178/540(33.0%) | | ACR |  |  | 7 | M |
| (Li et al., 2014) China[38] | East Asia | cohort study | 2104 | (29.2,12.1) * | 466(22.1%) | NA | NA | NA | NA | Oral ulcer | 466(22.1%) | | ACR |  |  | 6 | M |
| (Teh et al., 2014) Malaysia[39] | Southeast Asia | retrospective cohort study | 633 | (29.7,12.9) * | 244(38.5%) | NA | NA | NA | NA | Oral ulcer (East Asian) | 85/260(33%) | | ACR |  |  | 6 | M |
|  |  |  |  |  |  |  |  |  |  | Oral ulcer (Malay) | 79/198(40%) | |  |  |  |  |  |
| (Fakhreldin et al., 2015) Egypt[40] | Africa | retrospective cohort study | 250 | (27.38,7.74) * | 121(48.4%) | NA | NA | NA | NA | Oral ulcer | 121(48.4%) | | ACR |  |  | 6 | M |
| (Jeleniewicz et al., 2015) Poland[41] | Europe | retrospective cohort study | 128 | 56.3 (46–69) years  Late-onset(≧50years) (n = 20)；  Adult-onset(<50years) (n = 108) | 43(33.6%) | NA | NA | NA | NA | Oral ulcers (Late-onset) | 6/20(30%) | | ACR,1997 |  |  | 5 | M |
|  |  |  |  |  |  |  |  |  |  | Oral ulcers (Adult-onset) | 37/108(34.3%) | |  |  |  |  |  |
| (Joo & Bae, 2015) Korea[42] | East Asia | cohort study | 161 | (27.5,10.4) * | 46(28.6%) | NA | NA | NA | NA | Oral ulcer | 46(28.6%) | | ACR |  |  | 7 | M |
| (Peñaranda-Parada et al., 2015) Colombia[43] | South America | cross-sectional study | 170 | Late-onset(≧50years); Adult-onset SLE (18-49years) | 49(28.8%) | NA | NA | NA | NA | Oral ulcers (Late-onset) | 26/98(26.53%) | | ACR | 7 | M |  |  |
|  |  |  |  |  |  |  |  |  |  |  |  |  |  |  |  |  |  |
|  |  |  |  |  |  |  |  |  |  | Oral ulcers (Adult-onset) | 23/72(31.94%) | |  |  |  |  |  |
|  |  |  |  |  |  |  |  |  |  |  |  |  |  |  |  |  |  |
| (Ambrose et al., 2016) UK[44] | Europe | cohort study | 924 | Children-onset (0–17 years) ；  Adult-onset SLE (18-48years);  Late-onset (≧50years) (n=44) | 294/924(31.8%) | NA | NA | NA | NA | Oral ulcers (Children-onset) | 161/413(39%) | | ACR |  |  | 5 | M |
|  |  |  |  |  |  |  |  |  |  | Oral ulcers (Adult-onset) | 121/467(25.9%) | |  |  |  |  |  |
|  |  |  |  |  |  |  |  |  |  | Oral ulcers (Late-onset) | 14/44(31.8%) | |  |  |  |  |  |
| (Rasheed et al., 2016) Pakistan[45] | South Asia | cohort study | 125 | (31，11.2)* | 39（31.2%） | NA | NA | NA | NA | Oral ulcer | 39（31.2%） | | ACR |  |  | 7 | M |
| (Artim-Esen et al., 2017) Turkey[46] | West Asia | combined longitudinal cohort | 935 | Children-onset:(13.7,3.5) * years;  Adult-onset SLE:(34,11.3) * years | 161/935(17.2%) | NA | NA | NA | NA | Oral ulcers (Children-onset) | 50/216(23.1%) | | ACR |  |  | 6 | M |
|  |  |  |  |  |  |  |  |  |  | Oral ulcers (Adult-onset) | 111/719(15.4%) | |  |  |  |  |  |
| (Budhoo et al., 2017) South Africa[47] | Africa | retrospective cohort study | 408 | Adult-onset(<50years) :(28.71,9.87) * years(n=338);  Late-onset (≧50years) :(56.78,5.72) * years(n=59) | 204/408(50.0%) | NA | NA | 19/36(52.7%) | 185/372(49.7%) | Oral ulcer(Adult-onset) | 173/338(51.2%) | | ACR |  |  | 7 | M |
|  |  |  |  |  |  |  |  |  |  | Oral ulcer(Late-onset) | 27/59(45.8%) | |  |  |  |  |  |
|  |  |  |  |  |  |  |  |  |  | Oral ulcer(female) | 185/372(49.7%) | |  |  |  |  |  |
|  |  |  |  |  |  |  |  |  |  | Oral ulcer(male) | 19/36(52.8%) | |  |  |  |  |  |
|  |  |  |  |  |  |  |  |  |  | Oral ulcer(Indian) | 132/237(56%) | |  |  |  |  |  |
|  |  |  |  |  |  |  |  |  |  | Oral ulcer(Black race) | 52/137(38%) | |  |  |  |  |  |
|  |  |  |  |  |  |  |  |  |  | Oral ulcer(Caucasians) | 13/17(76%) | |  |  |  |  |  |
| (Maloney et al., 2017) Jamaica[48] | North America | cohort study | 150 | (33.2,10.9) * | 43/150(28.7%) | NA | NA | NA | NA | Oral ulcer | 43/150(28.7%) | | ACR |  |  | 7 | M |
| (Morais & Isenberg, 2017)UK[49] | Europe | cohort study | 620 | NA | 160/620（25.8%） | NA | NA | NA | NA | Oral ulcer (Caucasians) | 97/369（26%） | | ACR |  |  | 7 | M |
|  |  |  |  |  |  |  |  |  |  | Oral ulcer (East Asian) | 17/77(22%) | |  |  |  |  |  |
| (Sassi et al., 2017) Brazil[50] | South America | cross-sectional study | 598 | (33.6,14.3) * | 216/598(36.1%) | NA | NA | NA | NA | Oral ulcer | 216/598(36.1%) | | ACR | 6 | M |  |  |
| (Aggarwal et al., 2018) India[51] | South Asia | retrospective cohort study | 273 | median (range)14(1-17) | 63/273(23.0%) | NA | NA | NA | NA | Oral ulcer | 63/273(23.0%) | | ACR |  |  | 6 | M |
| (Dowaiki et al., 2018) Pakistan[52] | South Asia | retrospective cross-sectional | 285 | median (range)33(14-69) | 34/285(11.9%) | NA | NA | NA | NA | Oral ulcer | 34/285(11.9%) | | SLICC, 2012 | 5 | M |  |  |
| (Fedrigo et al., 2018) Brazil[53] | South America | retrospective cohort study | 455 | median(range) (IQR)4131.0–51.0 years) | 190/455(41.7%) | NA | NA | NA | NA | Oral ulcer | 190/455(41.7%) | | ACR |  |  | 6 | M |
| (Fonseca et al., 2018) Portugal[54] | Europe | cross-sectional study | 204 | Children-onset(≤16years) :(13.2,3.4) * years (n = 38);  Adult-onset SLE（>16years）:(34.2,12) * years(n=166) | 46/204(22.5%) | NA | NA | NA | NA | Oral ulcers (Children-onset) | 17/38(45.5%) | | ACR | 7 | M |  |  |
|  |  |  |  |  |  |  |  |  |  | Oral ulcers (Adult-onset) | 29/166(17.5%) | |  |  |  |  |  |
| (Mahmoud et al., 2018) Egypt[55] | Africa | retrospective cohort study | 770 | (22.1,8.6) * | 404(52.5%) | NA | NA | NA | NA | Oral ulcer | 404(52.5%) | | SLICC |  |  | 7 | M |
| (Manzano-Gamero et al., 2018) Spain[56] | Europe | cross-sectional study | 150 | （40.2，12.5）* | 58（38.7%） | NA | NA | NA | NA | Oral ulcer (Caucasians) | 34/98（35%） | | ACR | 8 | H |  |  |
| (Wei et al., 2018) China[57] | East Asia | retrospective case-control study | 190 | （31.89，11.56）* | 36（18.95%） | NA | NA | NA | NA | Oral ulcer | 36/190（18.95%） | | ACR |  |  | 8 | H |
| (Chen et al., 2019) China[58] | East Asia | retrospective cohort study | 407 | （34.8，14.0）* | 44(10.9%) | NA | NA | NA | NA | Oral ulcer | 44(10.9%) | | SLICC, 2012 |  |  | 7 | M |
| (Gergianaki et al., 2019) Greece[59] | Europe | cross-sectional study | 399 | NA | 199(49.9%) | NA | NA | NA | NA | Oral ulcer | 199(49.9%) | | ACR,1997 | 6 | M |  |  |
| (Hamijoyo et al., 2019) Indonesia[60] | Southeast Asia | retrospective cohort study | 813 | (27.7,9.4) * | 465 (57.2%) | NA | NA | 24/36 (66.7%) | 441/777 (56.8%) | Oral ulcer（Females） | 441/777(56.8%) | | ACR, 1982 |  |  | 7 | M |
|  |  |  |  |  |  |  |  |  |  | Oral ulcer（males） | 24/36(66.7%) | |  |  |  |  |  |
| (Ahn et al., 2020) Korea[61] | East Asia | retrospective cohort study | 171 | median (range):36.0 (26.3–46.0)  median(range):36.0 (26.3–46.0) | 21（12.3%） | NA | NA | NA | NA | Oral ulcer | 21（12.3%） | | ACR,1997 |  |  | 7 | M |
| (Saeed et al., 2021) Egypt[62] | Africa | cross-sectional study | 189 | (30.5,9.7) * | 105(55.6%) | NA | NA | 5/7(71.4%) | 100/182(54.9%) | patches | 100/189(53%) | | ACR | 6 | M |  |  |
|  |  |  |  |  |  |  |  |  |  | Oral candidiasis | 78/189(41.3%) | |  |  |  |  |  |
|  |  |  |  |  |  |  |  |  |  | Oral ulcer | 29/189(15.2%) | |  |  |  |  |  |
| (Chebbi et al., 2020) Tunisia[63] | Africa | descriptive cohort study | 253 | (30.8,11.8) * | 40(15.8%) | NA | NA | NA | NA | Oral ulcer | 40(15.8%) | | ACR |  |  | 7 | M |
| (Lim et al., 2020) Malaysia[64] | Southeast Asia | retrospective cohort study | 141 | median (range):10.8(9.0-12.0) | 69(48.9%) | NA | NA | NA | NA | Oral ulcer | 69(48.9%) | | ACR |  |  | 7 | M |
| (Nikolopoulos et al., 2020) Greece[65] | Europe | retrospective cohort study | 555 | (38.3,15.6) * | 98(17.7%) | NA | NA | NA | NA | Oral ulcer | 98(17.7%) | | ACR,1997 |  |  | 5 | M |
| (Prevete et al., 2020) Spain[66] | Europe | prospective cohort study | 395 | (44.2,15.6) * | 90(22.8%) | NA | NA | NA | NA | Oral ulcer | 90(22.8%) | | ACR,1997 |  |  | 5 | M |
| (Talukdar et al., 2020) India[67] | South Asia | cross-sectional study | 145 | (23.5,8.1) * | 37(25.5%) | NA | NA | NA | NA | Oral ulcer | 37(25.5%) | | ACR | 6 | M |  |  |
| (Chanprapaph et al., 2021)Thai[68] | Southeast Asia | retrospective cross-sectional study | 1006 | Adult-onset:(29.9,10.3) * years(n=740);  Late-onset(>50years) :(60.7,8.5) * years(n=266) | 251(24.95%) | NA | NA | NA | NA | Oral ulcer (Adult-onset) | 209/740(28.2%) | | ACR 1997 and/ or SLICC 2012 | 7 | M |  |  |
|  |  |  |  |  |  |  |  |  |  | Oral ulcer (Late-onset) | 42/266(15.8%) | |  |  |  |  |  |
| (Esquivel-Pedraza et al., 2021) Mexico[69] | North America | cross-sectional study | 181 | median (range):37(16-76) | 34(18.8%) | NA | NA | NA | NA | telangiectasia | 14(7.7%) | | ACR | 6 | M |  |  |
|  |  |  |  |  |  |  |  |  |  | ulcer | 6(3.3%) | |  |  |  |  |  |
|  |  |  |  |  |  |  |  |  |  | white reticular patch | 7(3.9%) | |  |  |  |  |  |
|  |  |  |  |  |  |  |  |  |  | erythematous plaque | 7(3.9%) | |  |  |  |  |  |
| (Cervera et al., 2009)UK[70] | Europe | prospective cohort study | 2840 | NA | 275(9.7%) | NA | NA | NA | NA | Oral ulcer | 275(9.7%) | | ACR |  |  | 3 | L |
| (Correa-Rodríguez et al., 2021) Spain[71] | Europe | cross-sectional study | 293 | （46.87，12.94 ）* | 145（52.0%） | NA | NA | NA | NA | Oral ulcer | 145（52.0%） | | SLICC/ACR | 2 | L |  |  |
| (Chottawornsak, N., et al.,2018) Thailand[72] | Southeast Asia | retrospective cohort study | 377 | Children-onset(≤18years) :(11.58,3.60) * years (n = 171);  Adult-onset SLE（>18years）:(32.76,10.17) * years(n=206) | 118(31.3%) | NA | NA | NA | NA | Oral ulcers (Children-onset) | 62/171 (36.3%) | | SLICC 2012 |  |  | 7 | M |
|  |  |  |  |  |  |  |  |  |  | Oral ulcers (Adult-onset) | 56/206 (27.2%) | |  |  |  |  |  |
| (Uthman, Imad, et al.,1999) Lebanon[73] | West Asia | retrospective cohort study | 100 | median age(y):25 | 40(40%) | NA | NA | NA | NA | Oral ulcer | 40/100(40%) | | ACR 1982 |  |  | 7 | M |
| (Ramírez Sepúlveda, Jorge I., et al.,2019)Sweden[74] | Europe | cohort study | 1226 | Women :(36,15)*(n=1060)  Men :(40,19)*(n=166) | 288(23.5%) | NA | NA | (26/166)15.7% | (262/1060) 24.7% | Oral ulcer | 288/1226(23.5%) | | ACR 1982/Fries agnostic  principle |  |  | 7 | M |
| (Jasmin, R., et al. ,2013)Malaysia[75] | Southeast Asia | cross-sectional study | 155 | (28.9, 12.4)* | 79(51%) | NA | NA | NA | NA | Oral ulcer（Chinese） | 41/85(48.2%) | | ACR | 8 | H |  |  |
|  |  |  |  |  |  |  |  |  |  | Oral ulcer（Malay） | 27/52（51.9 %） | |  |  |  |  |  |
|  |  |  |  |  |  |  |  |  |  | Oral ulcer (Indian) | 11/18（61.1%） | |  |  |  |  |  |
| (Aleem, Aamer, et al. ,2015) Saudi Arabia[76] | West Asia | cohort study | 624 | (34.3,11.9)* | 244 (39.1%) | NA | NA | NA | NA | Oral ulcer | 244/624(39.1%) | | ACR |  |  | 8 | H |
| (De Oliveira, Natália Teixeira, et al.,2020)Brazil[77] | South America | retrospective cohort study | 559 | median (range):29.0 (21.0-39.0) | 238/559 (42.5%) | NA | NA | 9/45 (20%) | 229/514 (44.5%) | Oral ulcer | 238/559 (42.5%) | | SLICC 2012 |  |  | 8 | H |
| (Alballa, S. R. ,1995) Saudi Arabia[78] | West Asia | cohort study | 87 | (25.3,10.5)* | 16 (18%) | NA | NA | NA | NA | Oral ulcer | 16 (18%) | | the  1982 revised ARA criteria |  |  | 7 | M |
| (Abid, N.,2013) Saudi Arabia[79] | West Asia | cohort study | 46 | (26.17 ,9.17)* | 33(71.7%) | NA | NA | NA | NA | Oral ulcer | 33(71.7%) | | ACR 1997 |  |  | 6 | M |
| (Novak, G. V., et al.,2018) Brazil[80] | South America | retrospective multicenter cohort study | 1555 | Childhood-onset(≤18years) | 533(34.3%) | NA | NA | NA | NA | Oral ulcer | 533(34.3%) | | ACR/SLICC |  |  | 5 | M |
| (QAMAR, SOBIA, et al.,2021) Pakistan[81] | South Asia | cohort study | 75 | (10.89,3.4)* | 56(74.7%) | NA | NA | NA | NA | Oral ulcer | 56(74.7%) | | ACR/SLICC |  |  | 6 | M |
| (AL-RAWI Z,1983) Iraq[82] | West Asia | prospective cohort study | 67 | 24.3 (10-48)* | 35(52%) | NA | NA | NA | NA | Oral ulcer | 35(52%) | | the A.R.A. criteria (1971) |  |  | 7 | M |
| (Al-Jarallah, K., et al.,1998) Kuwait[83] | West Asia | cohort study | 108 | median:31.5y | 36(33%) | NA | NA | NA | NA | Oral ulcer | 36(33%) | | ACR 1982 |  |  | 7 | M |
| (Antolin, J., et al.,1995) Spain[84] | Europe | cohort study | 194 | (34.7,13.9)* | 58(30%) | NA | NA | NA | NA | Oral ulcers (Children-onset)(<20y)(n=31) | 15/31(48%) | | ARA1982 |  |  | 7 | M |
|  |  |  |  |  |  |  |  |  |  | Oral ulcers (Adult-onset)(20-50)y  (n=134) | 33/134(25%) | |  |  |  |  |  |
|  |  |  |  |  |  |  |  |  |  | Oral ulcers (late-onset)(>50y)(n=29) | 10/29(34%) | |  |  |  |  |  |
| (Burge, S. M., et al.,1989) UK[85] | Europe | cross-sectional study | 53 | Male:43(27-71)*  Female:42(13-83)* | NA | NA | NA | NA | NA | Cheilitis | 3/53(6%) | | ARA | 6 | M |  |  |
|  |  |  |  |  |  |  |  |  |  | Oral ulcers | 19/53(36%) | |  |  |  |  |  |
|  |  |  |  |  |  |  |  |  |  | Chronic plaques | 2/53(4%) | |  |  |  |  |  |
| (Mumtaz, Shamaila, et al.,2020) Pakistan[86] | South Asia | cross-sectional study | 75 | range>16y | 34(45.3%) | NA | NA | NA | NA | Oral ulcers | 34/75(45.3%) | | ACR1997 | 8 | H |  |  |
| (Johnson, A. E., et al.,1994) Sweden/England/Brazil[87] | Europe/South America | cross-sectional study | 209 | NA | Oral ulcers (Europe)57/176(32%) | NA | NA | NA | NA | Oral ulcers (England) | 50/112(45%) | | ARA | 8 | H |  |  |
|  |  |  |  |  |  |  |  |  |  | Oral ulcers (Brazil) | 5/33(15%) | |  |  |  |  |  |
|  |  |  |  |  | Oral ulcers (South America)5/33(15%) |  |  |  |  | Oral ulcers (Sweden) | 7/64(11%) | |  |  |  |  |  |
| (Kudsi, Mayssoun, et al.,2021) Syria[88] | West Asia | cross-sectional study | 60 | NA | 42/60(70%) | NA | NA | 38/54(70.4%) | 4/6(66.7%) | Oral ulcers | 12/60 (20.0%) | | ACR2012 | 6 | M |  |  |
|  |  |  |  |  |  |  |  |  |  | erythema with a white centre | 7/60(11.7%) | |  |  |  |  |  |
|  |  |  |  |  |  |  |  |  |  | only erythema | 5/60(8.3%) | |  |  |  |  |  |
|  |  |  |  |  |  |  |  |  |  | white plaques | 1/60(1.7%) | |  |  |  |  |  |
| (Tomic-Lucic, Aleksandra, et al.,2013)Serbia[89] | Europe | cohort study | 60 | Late-onset:(56.93,6.93)*n=30  Adult-onset:(33.80,8.21)*n=30 | 5(8.3%) | NA | NA | NA | NA | ulcer(Late-onset) | 2/30(6.6%) | | ACR1997 |  |  | 7 | M |
|  |  |  |  |  |  |  |  |  |  | ulcer(adult-onset) | 3/30(10%) | |  |  |  |  |  |
| (Pradhan,2011) India[90] | South Asia | cross-sectional study | 80 | (27.5,9.52)* | 18(22.5%) | NA | NA | NA | NA | Oral ulcers | 18(22.5%) | | ACR1997 | 6 | M |  |  |
| (Wang, H,2021) China[91] | East Asia | retrospective cohort study | 89 | median (IQR)40.5 (26.75–50.0) | 12（13.5%） | NA | NA | NA | NA | Oral ulcers | 12（13.5%） | | the 2010 revised Chinese Rheumatology Association (CRA) revised classification criteria |  |  | 6 | M |
| (Meyer, Ulrich, et al.2000) German[92] | Europe | cross-sectional study | 46 | (39.7,13)* | 22(48%) | NA | NA | NA | NA | Erythema | 9/46（19.6%） | | ACR | 7 | M |  |  |
|  |  |  |  |  |  |  |  |  |  | Ulcer/Aphthous ulcer | 18/46(39%) | |  |  |  |  |  |
| (Segasothy, M,2001) Australia[93] | Oceania | retrospective cohort study | 24 | NA | 6(25%) | NA | NA | NA | NA | oral ulcers（Aborigines） | 3/18(16.7%) | | ACR |  |  | 7 | M |
|  |  |  |  |  |  |  |  |  |  | oral ulcers（Caucasians） | 3/6(50%) | |  |  |  |  |  |
| (Feng, H,2006) China[94] | East Asia | cross-sectional study | 92 | 33(15-58)* | 25(27.1%) | NA | NA | NA | NA | Oral ulcers | 25(27.1%) | | ACR1982 | 7 | M |  |  |
| (Chen, T，2009)China[95] | East Asia | retrospective cohort study | 69 | Late-onset:(70.4,7.1)*n=19  Adult-onset:(27.2,6.5)*n=50 | 8(11.6%) | NA | NA | NA | NA | ulcer(Late-onset) | 5/19(26.3%) | | ACR1997 |  |  | 6 | M |
|  |  |  |  |  |  |  |  |  |  | ulcer(adult-onset) | 3/50(6.0%) | |  |  |  |  |  |
| (Shabana, A,2009) Egyp[96] | Africa | Case-control | 38 | (25.0,9.25)* | 12(31.6%) | NA | NA | NA | NA | Oral ulcers | 12(31.6%) | | ACR |  |  | 6 | M |
| (Fernandes, E,2010) Brazil[97] | South America | Case-control | 26 | Juvenile | 18(69.2%) | NA | NA | NA | NA | Oral ulcers(Child-onset) | 18(69.2%) | | ACR |  |  | 8 | H |
| (Gonçalves, L,2010) Brazil[98] | South America | cross-sectional study | 18 | range:(5-88) | 4(22%) | NA | NA | NA | NA | erythema | 3/18(16.7%) | | NA | 7 | M |  |  |
|  |  |  |  |  |  |  |  |  |  | oral ulcers | 1/18(5.6%) | |  |  |  |  |  |
| (Baştuǧ, F,2011)Turkey[99] | West Asia | retrospective cohort study | 30 | 12.3(3-6)* | 8(26.7%) | NA | NA | NA | NA | oral ulcers | 8/30(26.7%) | | ACR1982 |  |  | 8 | H |
| (Nakashima, et al.,2011) Brazil[100] | South America | cross-sectional study | 14 | (41.5,14.44)* | 3(21.4%) | NA | NA | NA | NA | oral ulcers | 3/14(21.4%) | | ACR | 5 | M |  |  |
| (Dönmez, S,2012) Turkey[101] | West Asia | cross-sectional | 78 | (39.8，11.2)* | 54（69.2%） | NA | NA | 0 | 54/78（69.2%） | oral ulcers | 54/78（69.2%） | | ACR | 6 | M |  |  |
| (Saigal, R，2012) India[102] | South Asia | cohort study | 60 | (28.1,11.2)* | 37(61.7%) | NA | NA | NA | NA | oral ulcers | 37/60(61.7%) | | ACR1982 |  |  | 7 | M |
| (Zaidan,2012) Iraq[103] | West Asia | cross-sectional | 50 | (32.24,9.26)* range:(16-53)y | NA | NA | NA | NA | NA | Oral ulcers | 36/50(72%) | | ACR1982 | 5 | M |  |  |
|  |  |  |  |  |  |  |  |  |  | Petechiae and purpura | 2/50(4%) | |  |  |  |  |  |
|  |  |  |  |  |  |  |  |  |  | Vesicles and Bullai | 5/50(10%) | |  |  |  |  |  |
| (Zakeri, Zahra,2012) Iran[104] | West Asia | retrospective cohort study | 13 | 34(12-52)* | 3(23.1%) | NA | NA | 0 | 3/13(23.1%) | oral ulcers | 3/13(23.1%) | | ACR |  |  | 5 | M |
| (Tareen, A,2014) Pakistan[105] | South Asia | cross-sectional | 82 | 34.91(8-62)* | 63(76.8%) | NA | NA | NA | NA | oral ulcers | 63/82(76.8%) | | ACR1997 | 6 | M |  |  |
| (Compagno, M,2016) Sweden[106] | Europe | cross-sectional | 69 | (41.4,13.7)* | 16(23.2%) | NA | NA | NA | NA | oral ulcers | 16(23.2%) | | ACR | 5 | M |  |  |
| (Ahadian, H，2017)Iran[107] | West Asia | cross-sectional | 8 | NA | 3/8(37.5%) | NA | NA | NA | NA | red/white plaques | 2/8(25%) | | NA | 5 | M |  |  |
|  |  |  |  |  |  |  |  |  |  | oral ulcers | 1/8(12.5%) | |  |  |  |  |  |
| (Hammoudeh,2018) Qatar[108] | West Asia | cross-sectional | 42 | (38.31,10.65)* | NA | NA | NA | NA | NA | oral ulcers | 3/42(7.1%) | | ACR1997 | 8 | H |  |  |
|  |  |  |  |  |  |  |  |  |  | Petechiae | 7/42(16.7%) | |  |  |  |  |  |
|  |  |  |  |  |  |  |  |  |  | Cheilitis | 1/42(2.4%) | |  |  |  |  |  |
|  |  |  |  |  |  |  |  |  |  | Candidiasis | 1/42(2.4%) | |  |  |  |  |  |
| (Crincoli, V,2020) Italy[109] | Europe | cross-sectional | 55 | (44.44,15.04)* | 29(52.7%) | NA | NA | NA | NA | Candidiasis(Caucasian) | 1(1.8%) | | NA | 5 | M |  |  |
|  |  |  |  |  |  |  |  |  |  | Cheilitis(Caucasian) | 5(9.1%) | |  |  |  |  |  |
|  |  |  |  |  |  |  |  |  |  | Erythema(Caucasian) | 5(9.1%) | |  |  |  |  |  |
|  |  |  |  |  |  |  |  |  |  | Petechiae(Caucasian) | 3(5.5%) | |  |  |  |  |  |
|  |  |  |  |  |  |  |  |  |  | Oral ulcers(Caucasian) | 11(20%) | |  |  |  |  |  |
| (Kumar, P,2020) India[110] | South Asia | cross-sectional | 27 | (29.7,10.7)* | 14(51%) | NA | NA | NA | NA | oral ulcers | 14(51%) | | ACR1997 | 9 | H |  |  |
| (Bongomin, F,2021) Uganda[111] | Africa | retrospective chart review | 56 | Median:29(14-65) | 23(41.1%) | NA | NA | NA | NA | oral ulcers | 23(41.1%) | | ACR1997, SLICC2012, ACR/EULAR |  |  | 7 | M |
| (Buonavoglia,2021) Italy[112] | Europe | pilot case–control | 16 | Median:39y | 6(37.5%) | NA | NA | NA | NA | oral ulcers | 6(37.5%) | | NA |  |  | 7 | M |
| (Manzano,2021) Brazil[113] | South America | case-control | 28 | (41.5,12.0)* | NA | NA | NA | NA | NA | candidiasis | 3 (11%) | | ACR |  |  | 6 | M |
|  |  |  |  |  |  |  |  |  |  | Leucoplakia | 1 (4%) | |  |  |  |  |  |
|  |  |  |  |  |  |  |  |  |  | Traumatic ulcer | 1 (4%) | |  |  |  |  |  |

age(years)*= mean (range) or mean (SD)(years); NA=none available; L=low quality; M= moderate quality; H=high quality; ACR: the American College of Rheumatology diagnostic criteria;ARA:the American Rheumatism Association;SLICC: the Systemic Lupus International Collaborating Clinics criteria

| **Variable** |  | **No. of Articles** |  | **No. of Participants** |  | **Heterogeneity Tests** | | |  | **effects model** |  | **Prevalence (95% CI)（%）** |  | **p-Value**  **(subgroup differences)** | | |
| --- | --- | --- | --- | --- | --- | --- | --- | --- | --- | --- | --- | --- | --- | --- | --- | --- |
|  |  |  |  |  |  | **p-Value** |  | **I^2^（%）** |  |  |  |  |  |  |  |  |
| **Sex** |  |  |  |  |  |  |  |  |  |  |  |  |  |  |  |  |
| male |  | 13 |  | 559 |  | <0.01 |  | 90 |  | random |  | 34(22;48) |  | 0.78 | | |
| female |  | 15 |  | 4419 |  | <0.01 |  | 97 |  | random |  | 37(27;47) |  |  |  |  |
| **Year of publication** |  |  |  |  |  |  |  |  |  |  |  |  |  |  |  |  |
| 2000-2009 |  | 15 |  | 8420 |  | <0.01 |  | 98 |  | random |  | 26(20;34) |  | 0.30 | | |
| 2010-2015 |  | 33 |  | 17870 |  | <0.01 |  | 97 |  | random |  | 35(28;41) |  |  |  |  |
| 2016-2022 |  | 54 |  | 26033 |  | 0 |  | 98 |  | random |  | 31(26;35) |  |  |  |  |
| Before 2000 |  | 7 |  | 811 |  | <0.01 |  | 79 |  | random |  | 35(27;43) |  |  |  |  |
| **Region** |  |  |  |  |  |  |  |  |  |  |  |  |  |  |  |  |
| East Asia |  | 19 |  | 12250 |  | <0.01 |  | 97 |  | random |  | 18(14;23) |  | <0.01* | | |
| West Asia |  | 16 |  | 3646 |  | <0.01 |  | 96 |  | random |  | 39(29;49) |  |  |  |  |
| South Asia |  | 12 |  | 1877 |  | <0.01 |  | 96 |  | random |  | 42(29;55) |  |  |  |  |
| Southeast Asia |  | 7 |  | 3236 |  | <0.01 |  | 97 |  | random |  | 40(31;49) |  |  |  |  |
| Europe |  | 28 |  | 17604 |  | 0 |  | 98 |  | random |  | 28(23;33) |  |  |  |  |
| North America |  | 4 |  | 7065 |  | <0.01 |  | 97 |  | random |  | 31(21;43) |  |  |  |  |
| South America |  | 13 |  | 5060 |  | <0.01 |  | 93 |  | random |  | 34(25;43) |  |  |  |  |
| Africa |  | 10 |  | 2372 |  | <0.01 |  | 97 |  | random |  | 37(27;47) |  |  |  |  |
| Oceania |  | 1 |  | 24 |  | NA |  | NA |  | NA |  | 25(10;47) |  |  | | |
| **Disease Status** |  |  |  |  |  |  |  |  |  |  |  |  |  |  |  |  |
| Active |  | 2 |  | 275 |  | 0.38 |  | 0 |  | common |  | 77(72;82) |  | <0.01* | | |
| inactive |  | 2 |  | 413 |  | <0.01 |  | 97 |  | random |  | 18(1;47) |  |  |  |  |
| **Sample size** |  |  |  |  |  |  |  |  |  |  |  |  |  |  | | |
| <100 |  | 28 |  | 1460 |  | <0.01 |  | 92 |  | random |  | 40(32;49) |  | 0.01* | | |
| ≧100 |  | 81 |  | 51674 |  | 0 |  | 99 |  | random |  | 29(26;32) |  |  |  |  |

1. **Subgroup analysis of OMLs prevalence among SLE patients. (Supplementary Table S3)**

**Table S3**. Subgroup analysis of OMLs prevalence among SLE patients.

* p < 0.05 (two-tailed).

| **Variable** |  | **No. of Articles** |  | **No. of Participants** |  | **Heterogeneity Tests** | | |  | **effects model** |  | **Prevalence (95% CI)（%）** |  | **p-Value**  **(subgroup differences)** | | |
| --- | --- | --- | --- | --- | --- | --- | --- | --- | --- | --- | --- | --- | --- | --- | --- | --- |
|  |  |  |  |  |  | **p-Value** |  | **I^2^（%）** |  |  |  |  |  |  |  |  |
| **Race** |  |  |  |  |  |  |  |  |  |  |  |  |  |  |  |  |
| East Asian |  | 4 |  | 680 |  | <0.01 |  | 92 |  | random |  | 29(17;43) |  | <0.01* | | |
| Blacks |  | 3 |  | 664 |  | 0.01 |  | 78 |  | random |  | 33(26;41) |  |  |  |  |
| Caucasians |  | 7 |  | 1474 |  | <0.01 |  | 92 |  | random |  | 41(27;55) |  |  |  |  |
| Malay |  | 2 |  | 250 |  | 0.12 |  | 58 |  | random |  | 44(33;56) |  |  |  |  |
| India |  | 2 |  | 255 |  | 0.68 |  | 0 |  | common |  | 56(50;62) |  |  |  |  |
| **Region** |  |  |  |  |  |  |  |  |  |  |  |  |  |  |  |  |
| East Asia |  | 19 |  | 12250 |  | <0.01 |  | 97 |  | random |  | 18(14;23) |  | <0.01* | | |
| West Asia |  | 19 |  | 3751 |  | <0.01 |  | 94 |  | random |  | 33(24;42) |  |  |  |  |
| South Asia |  | 12 |  | 1877 |  | <0.01 |  | 96 |  | random |  | 42(29;55) |  |  |  |  |
| Southeast Asia |  | 7 |  | 3236 |  | <0.01 |  | 97 |  | random |  | 40(31;49) |  |  |  |  |
| Europe |  | 28 |  | 16950 |  | <0.01 |  | 98 |  | random |  | 27(23;32) |  |  |  |  |
| North America |  | 4 |  | 7065 |  | <0.01 |  | 100 |  | random |  | 16(2;41) |  |  |  |  |
| South America |  | 12 |  | 5612 |  | <0.01 |  | 89 |  | random |  | 35(28;42) |  |  |  |  |
| Africa |  | 9 |  | 2359 |  | <0.01 |  | 97 |  | random |  | 33(23;44) |  |  |  |  |
| Oceania |  | 1 |  | 24 |  | NA |  | NA |  | NA |  | 25(10;47) |  |  |  |  |
| **Age** |  |  |  |  |  |  |  |  |  |  |  |  |  |  |  |  |
| Child-onset |  | 8 |  | 2680 |  | <0.01 |  | 87 |  | random |  | 41(32;50) |  | 0.02* | | |
| Adult-onset |  | 12 |  | 4014 |  | <0.01 |  | 95 |  | random |  | 26(19;33) |  |  |  |  |
| Late-onset |  | 8 |  | 565 |  | <0.01 |  | 79 |  | random |  | 26(17;36) |  |  |  |  |

1. **Subgroup analysis of oral ulcer prevalence among SLE patients. (Supplementary Table S4)**

**Table S4**. Subgroup analysis of oral ulcer prevalence among SLE patients.

* p < 0.05 (two-tailed).

1. **Prevalence of oral ulcers among SLE patients (Forest plot of the included studies). (Supplementary Figure S1)**

**
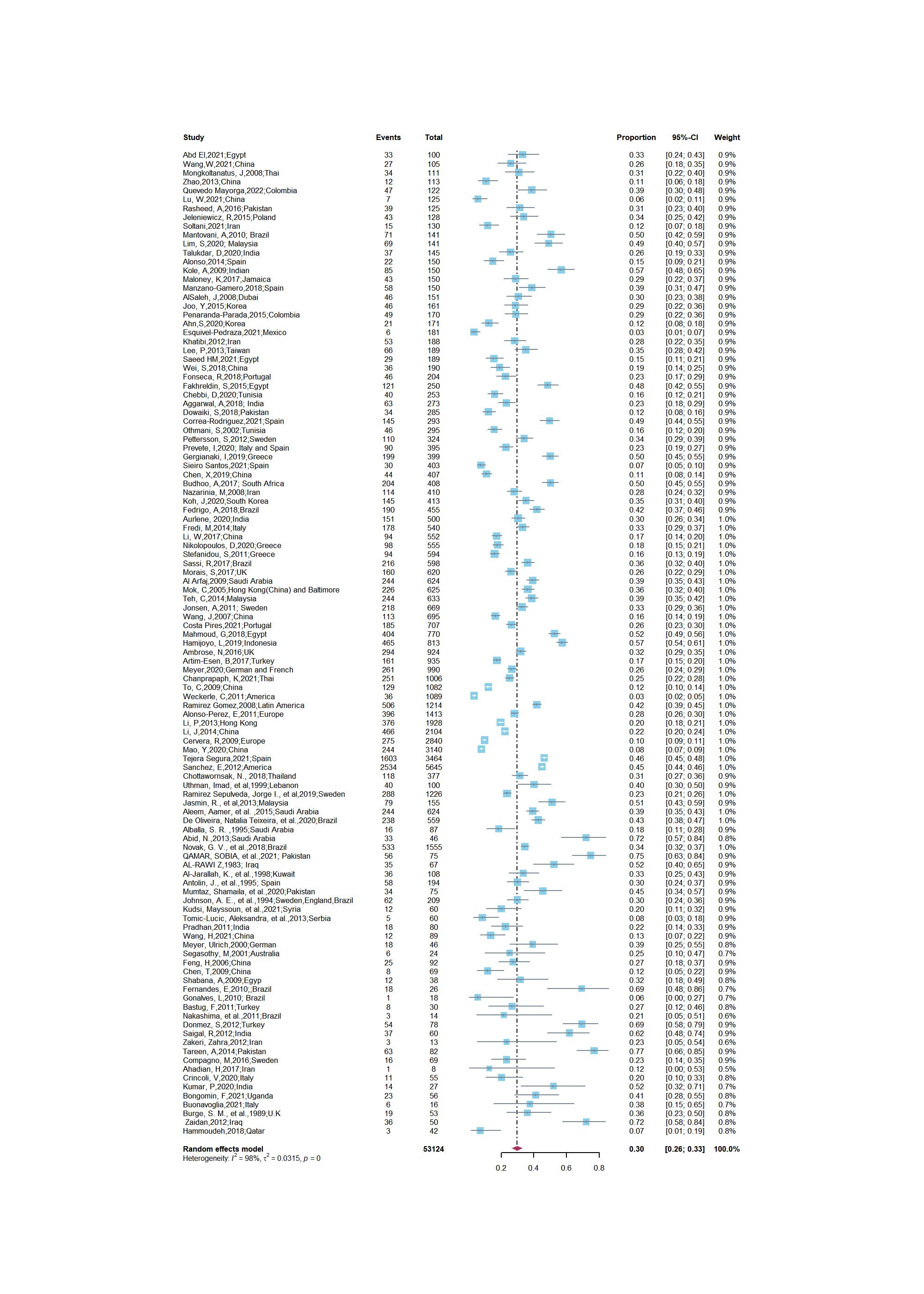
**

**Figure** **S1.** Prevalence of oral ulcers among SLE patients

1. **Oral mucosal diseases prevalence among SLE patients by sex. (Supplementary Figure S2)**


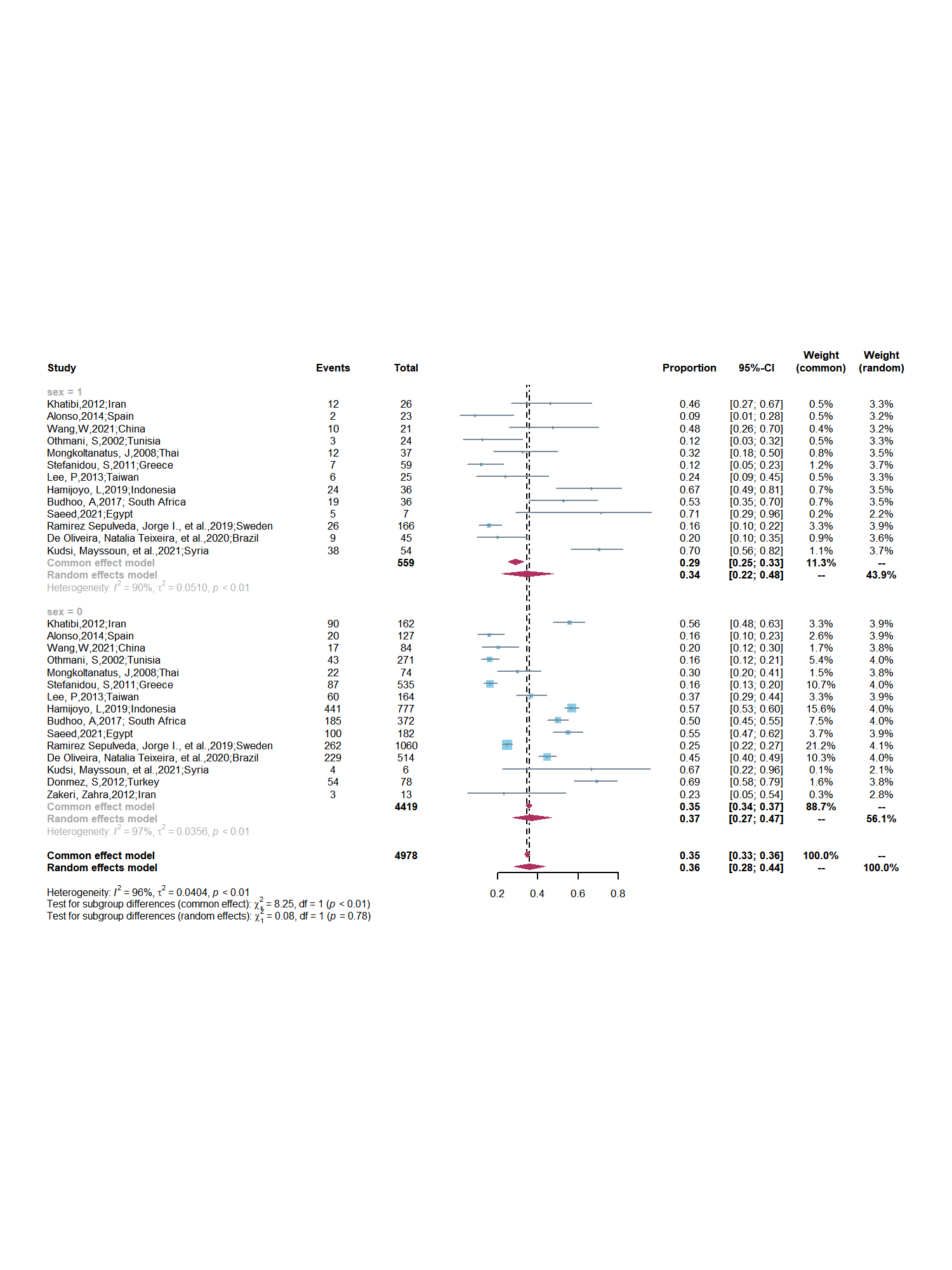


**Figure S2**. Oral mucosal diseases prevalence among SLE patients by sex

sex0=female; sex1=male

1. **Oral mucosal diseases prevalence among SLE patients by year of publication. (Supplementary Figure S3)**

**
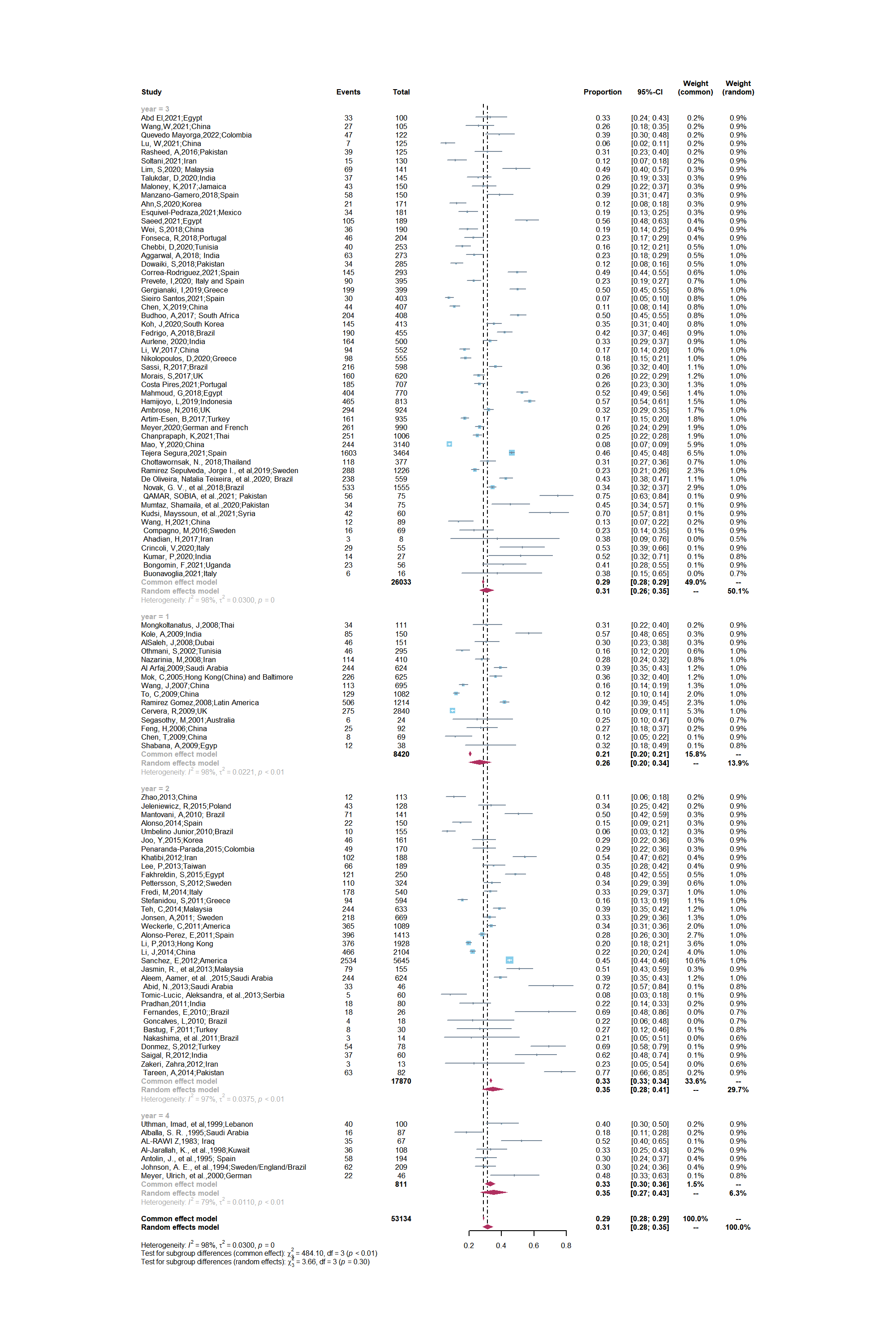
**

**Figure S3**. Oral mucosal diseases prevalence among SLE patients by year of publication

year=1 means Year of publication =2000-2009;

year=2 means Year of publication =2010-2015;

year=3 means Year of publication =2016-2022;

year=4 means Year of publication =before 2000.

1. **Oral mucosal diseases prevalence among SLE patients by region. (Supplementary Figure S4)**

**
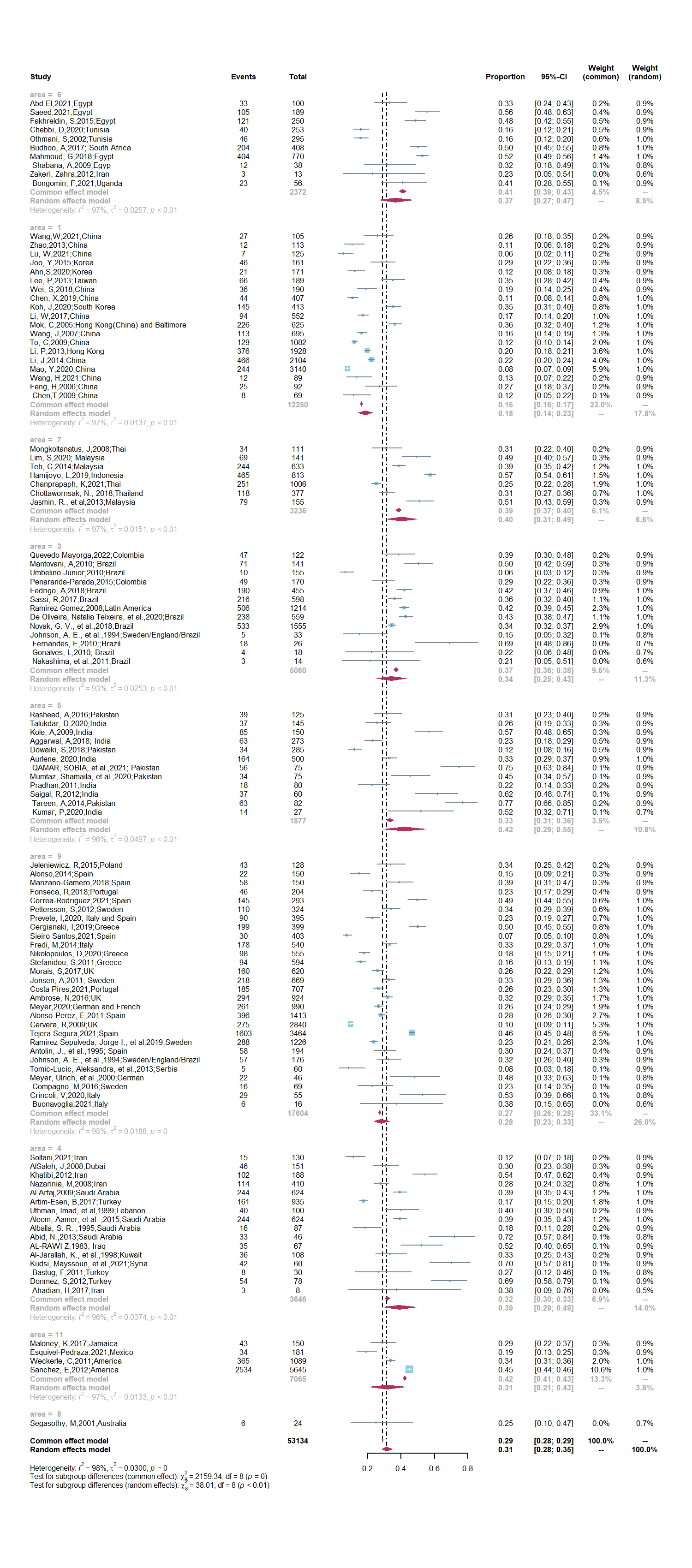
**

**Figure S4**. Oral mucosal diseases prevalence among SLE patients by region

area1=East Asia; area3= South America; area4= West Asia; area5= South Asia;

area6= Africa; area7= Southeast Asia; area8= Oceania; area9= Europe; area11= North America

1. **Oral mucosal diseases prevalence among SLE patients by disease status. (Supplementary Figure S5)**

**
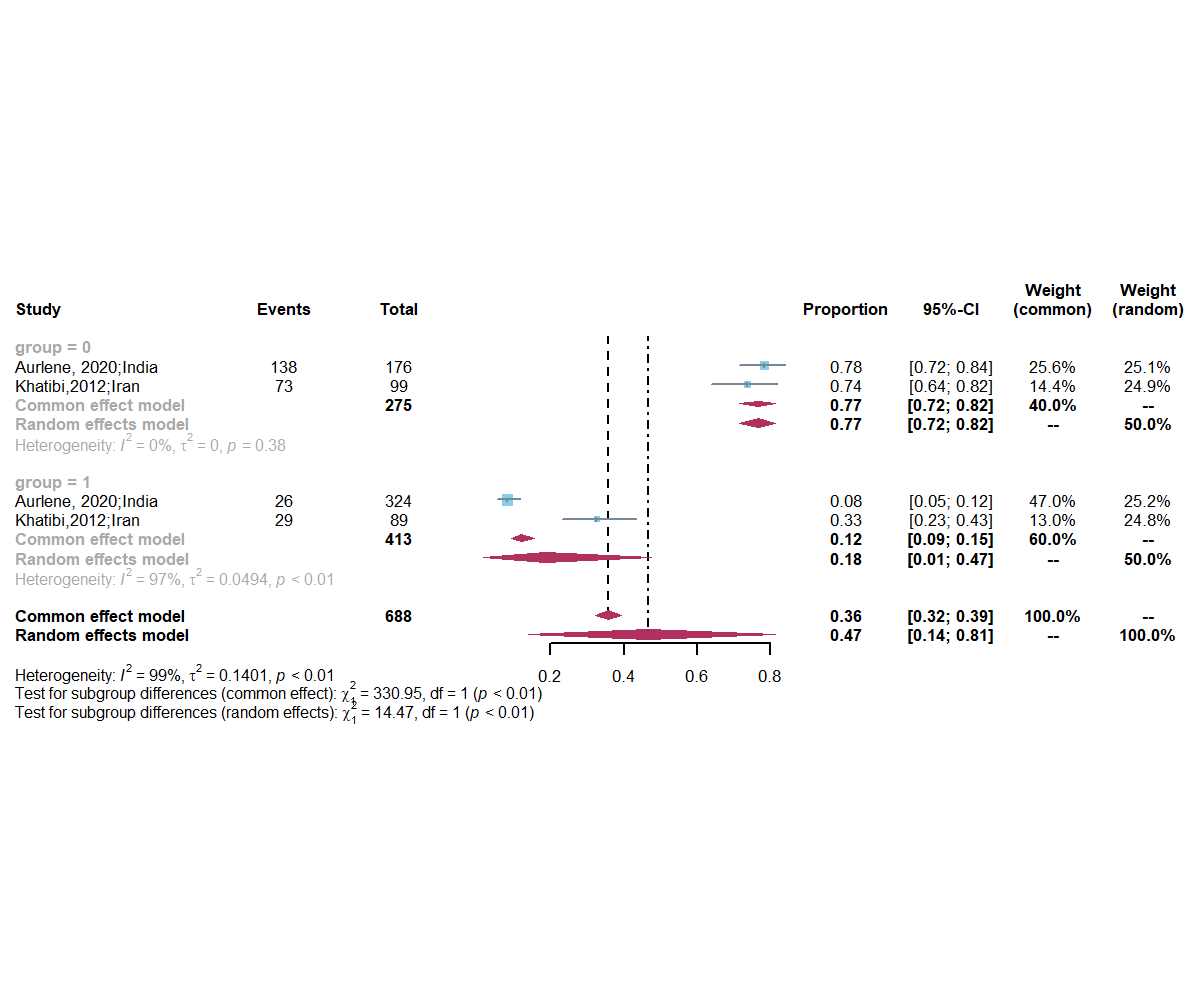
**

**Figure S5**. Oral mucosal diseases prevalence among SLE patients by disease status

group0=active; group1=inactive

1. **Oral mucosal diseases prevalence among SLE patients by** **sample size. (Supplementary Figure S6)**

**
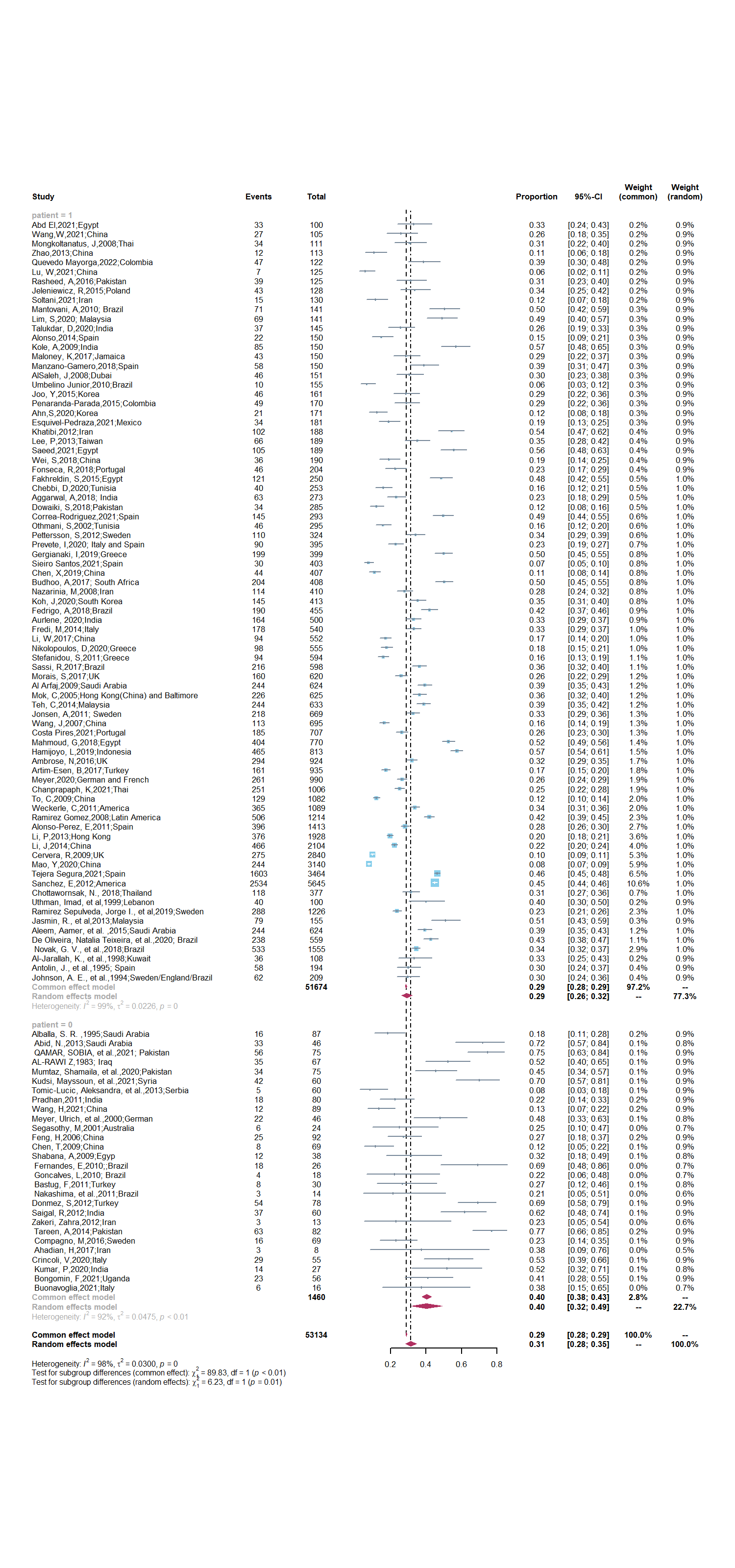
**

**Figure S6**. Oral mucosal diseases prevalence among SLE patients by sample size

group0 means n<100; group1 means n≧100

1. **Sensitivity Analysis（Supplementary Figure S7）**

**
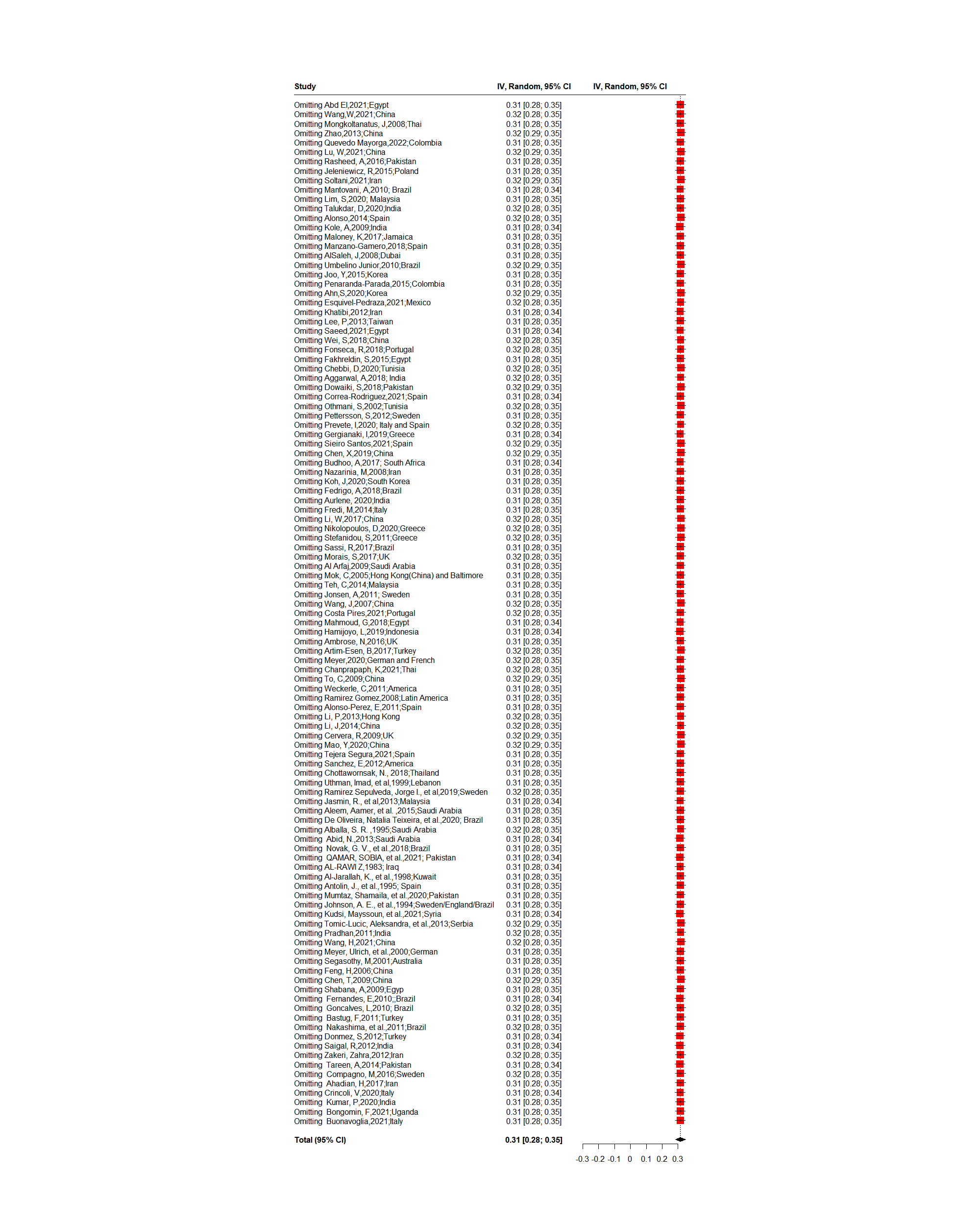
**

**Figure S7**. Sensitivity analysis of pooled studies in the meta-analysis on prevalence of oral mucosal disease among SLE patients.

1. **Publication Bias**


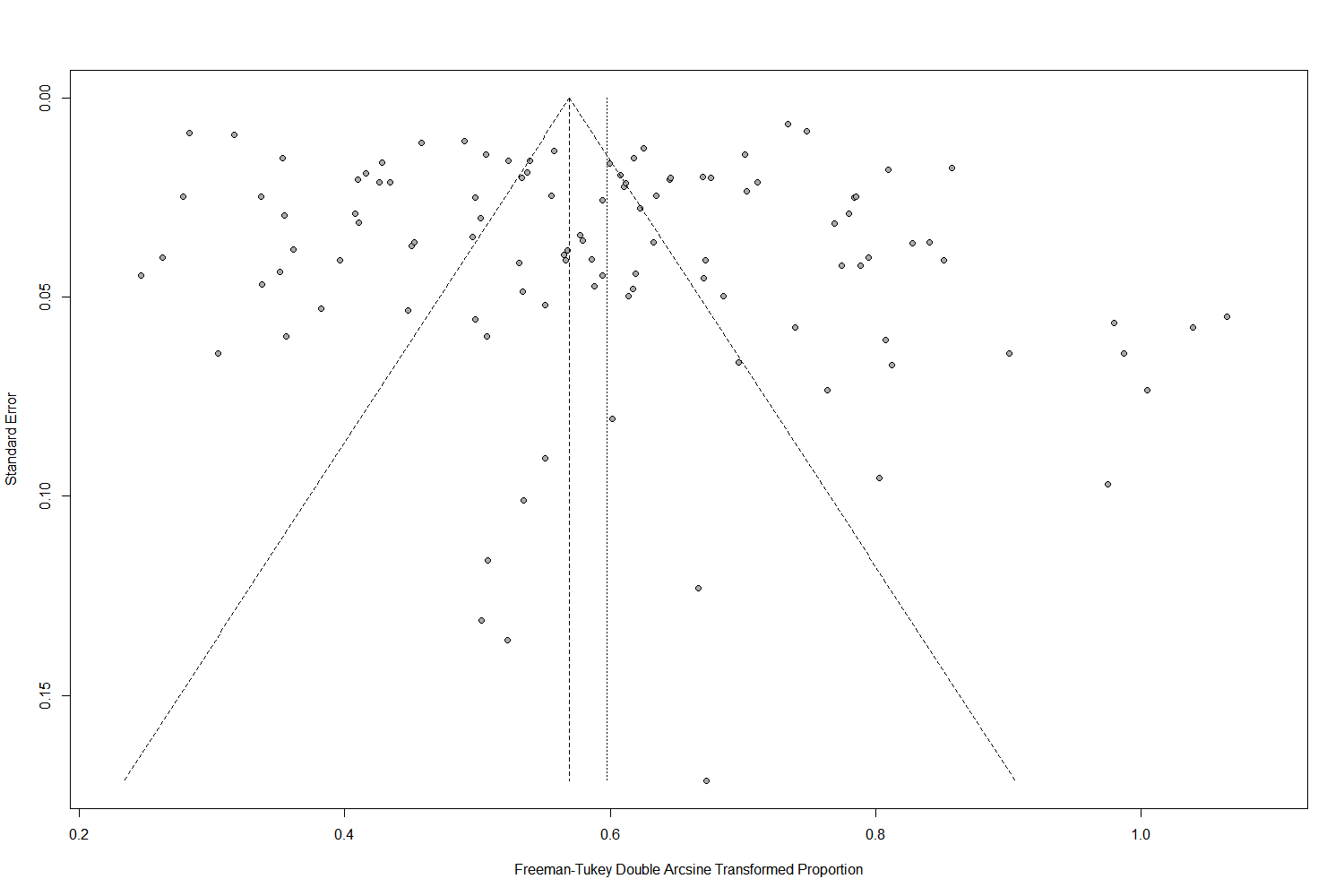


**Figure S8**. Funnel plot for studies reporting the prevalence of oral mucosal disease in SLE patients.

1. **Oral ulcers prevalence among SLE patients by age of onset. (Supplementary Figure S9)**

**
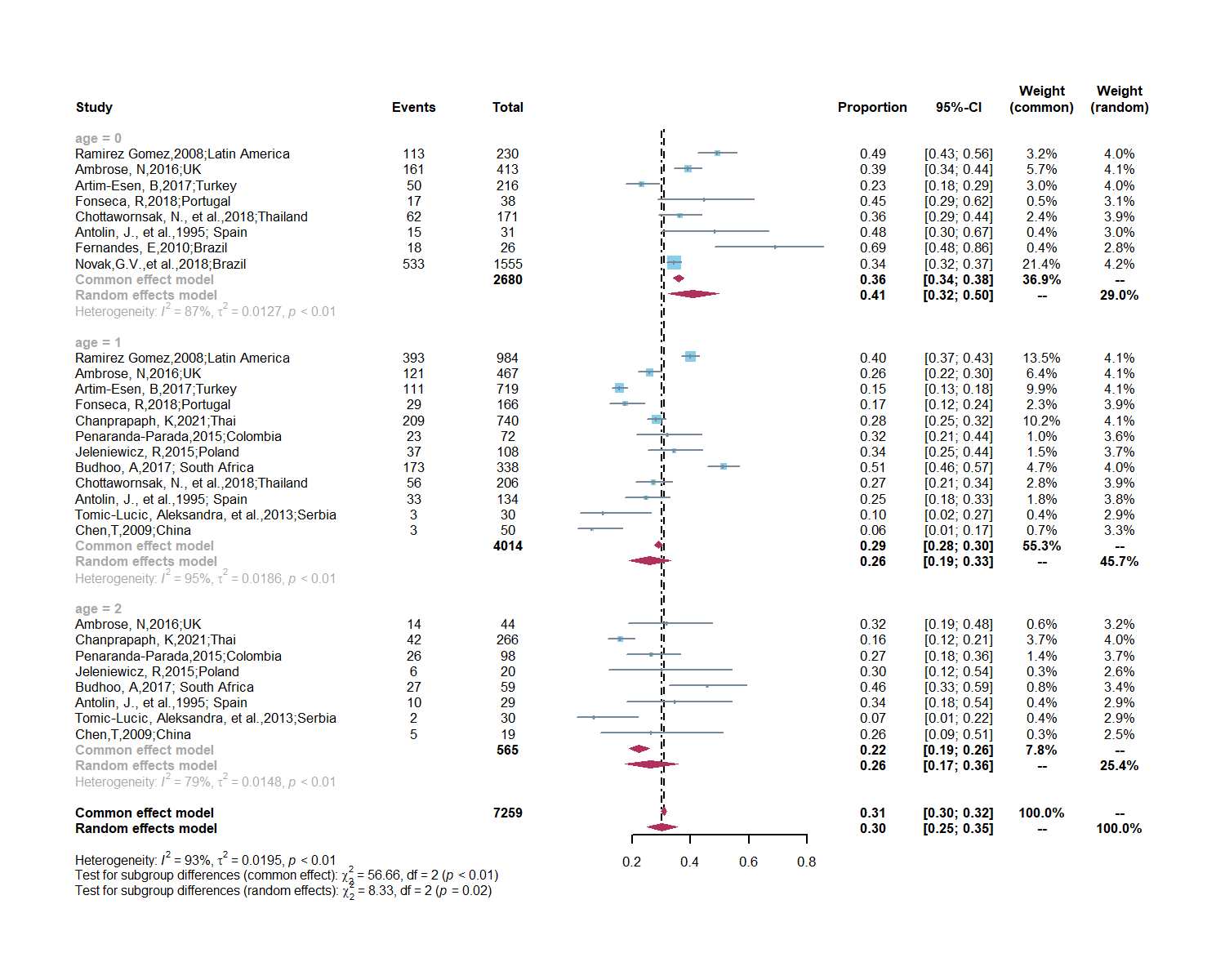
**

**Figure S9**. Oral mucosal diseases prevalence among SLE patients by age

age0= Child-onset; age1= Adult-onset; age2= Late-onset

1. **Oral ulcers prevalence among SLE patients by race. (Supplementary Figure S10)**


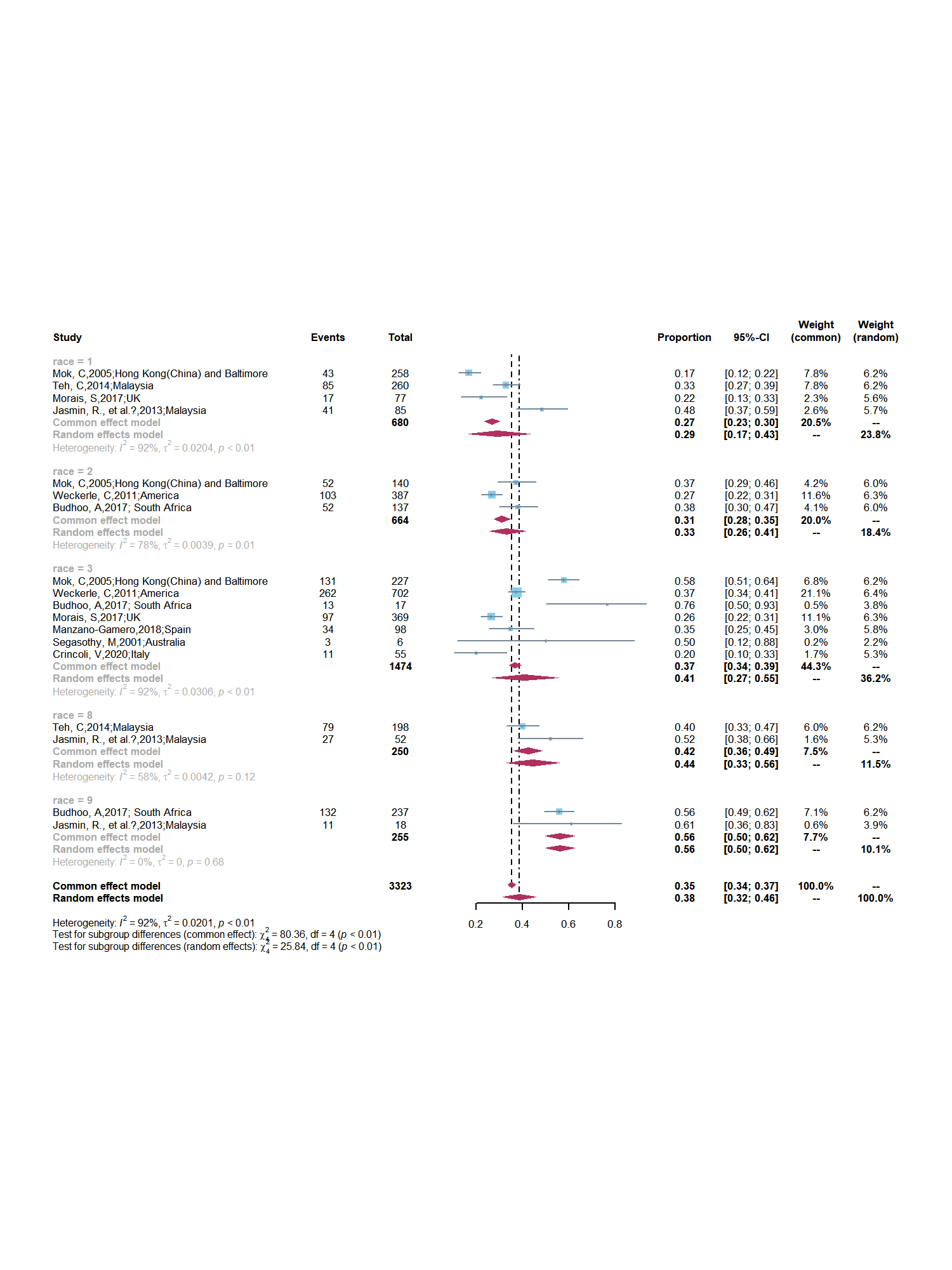


**Figure S10**. Oral mucosal diseases prevalence among SLE patients by race

race1=East Asian; race2=Blacks; race3=Caucasians; race8=Malay; race9=Indian

1. **Oral ulcers prevalence among SLE patients by region. (Supplementary Figure S11)**


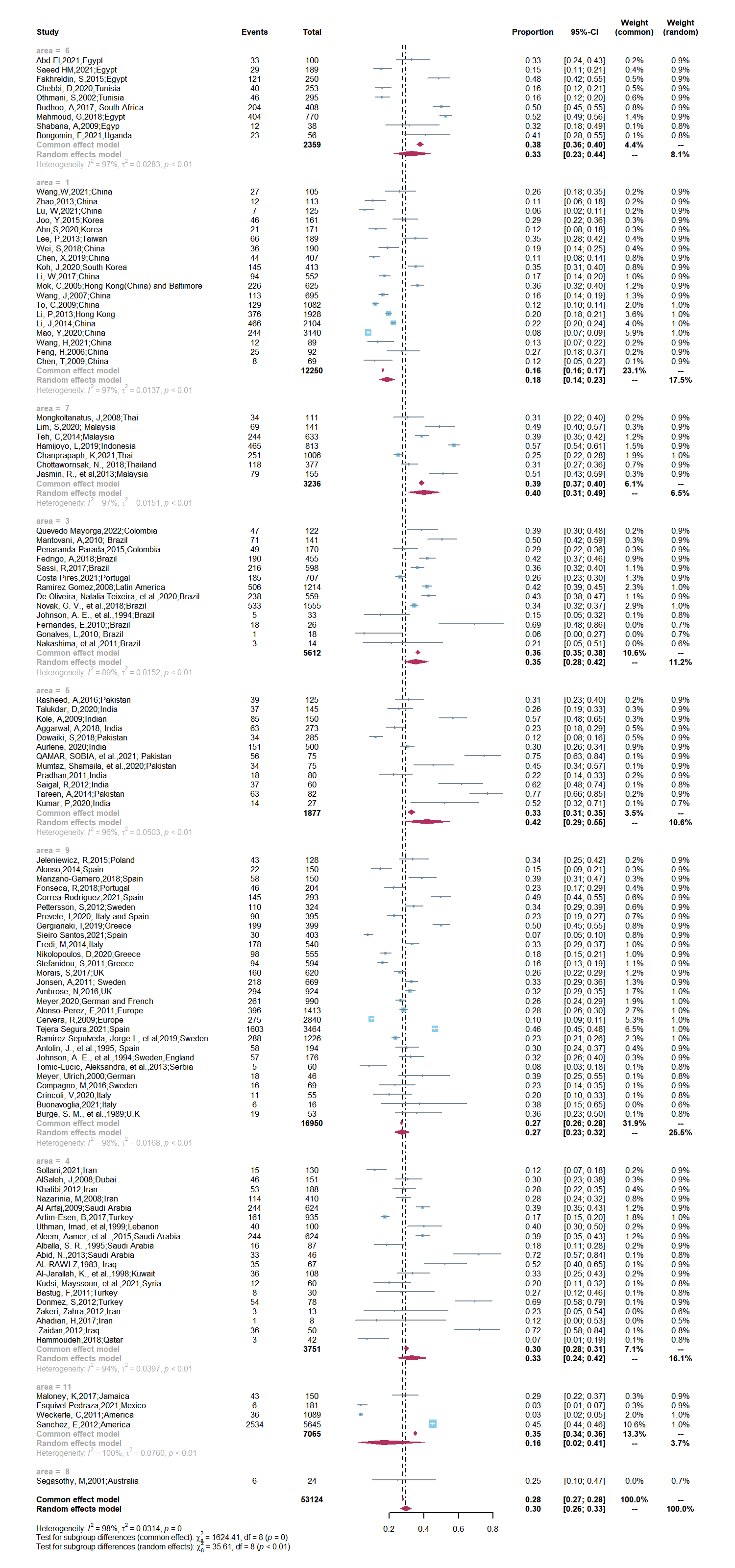


**Figure S11**. Oral ulcers prevalence among SLE patients by region

area1=East Asia; area3= South America; area4= West Asia; area5= South Asia;

area6= Africa; area7= Southeast Asia; area8= Oceania; area9= Europe; area11= North America

1. **Prevalence of erythema among SLE patients (Forest plot of the included studies). (Supplementary Figure S12)**


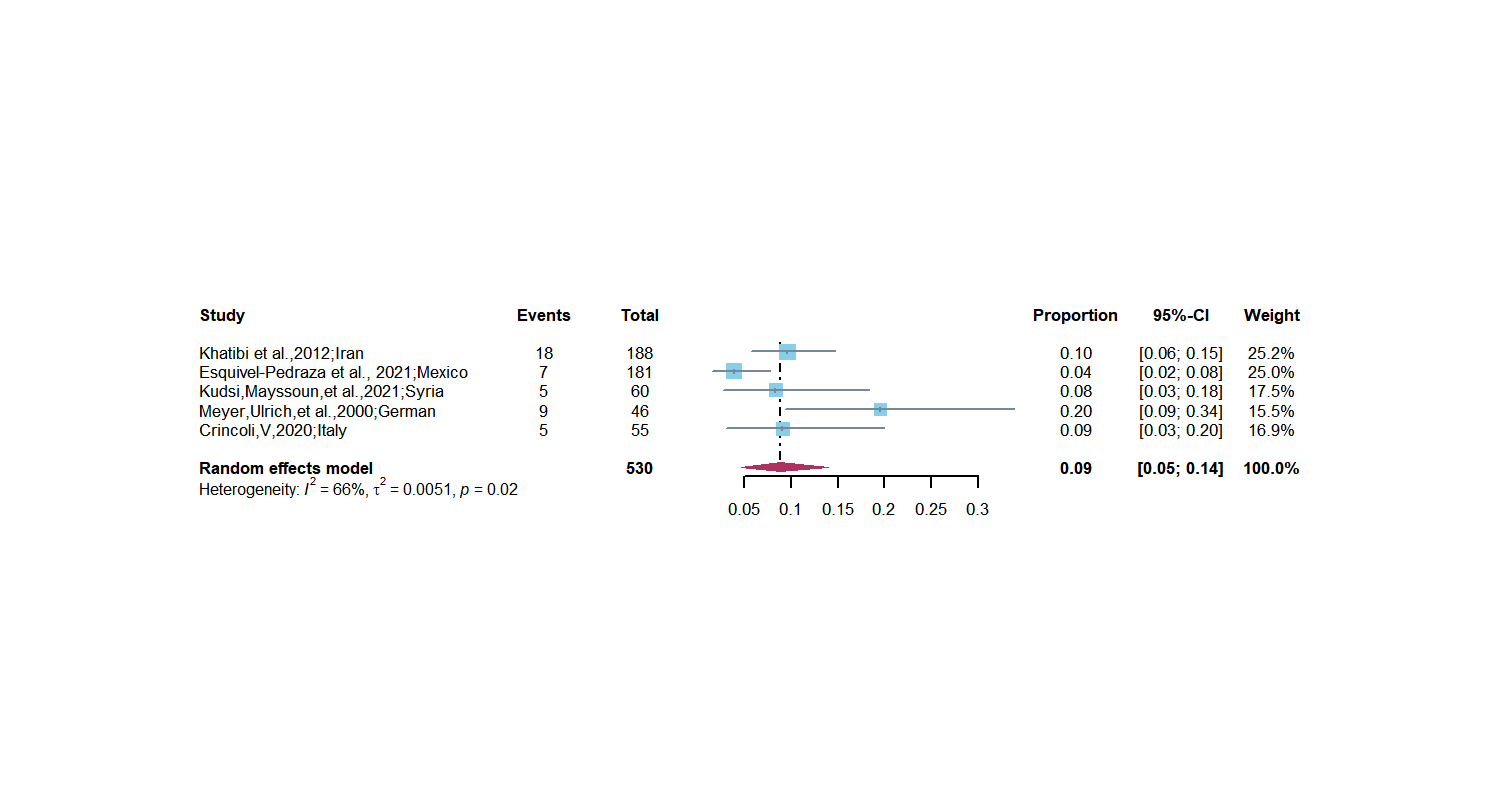


**Figure** **S12.** Prevalence of erythema among SLE patients

1. **Prevalence of white plaque among SLE patients (Forest plot of the included studies). (Supplementary Figure S13)**


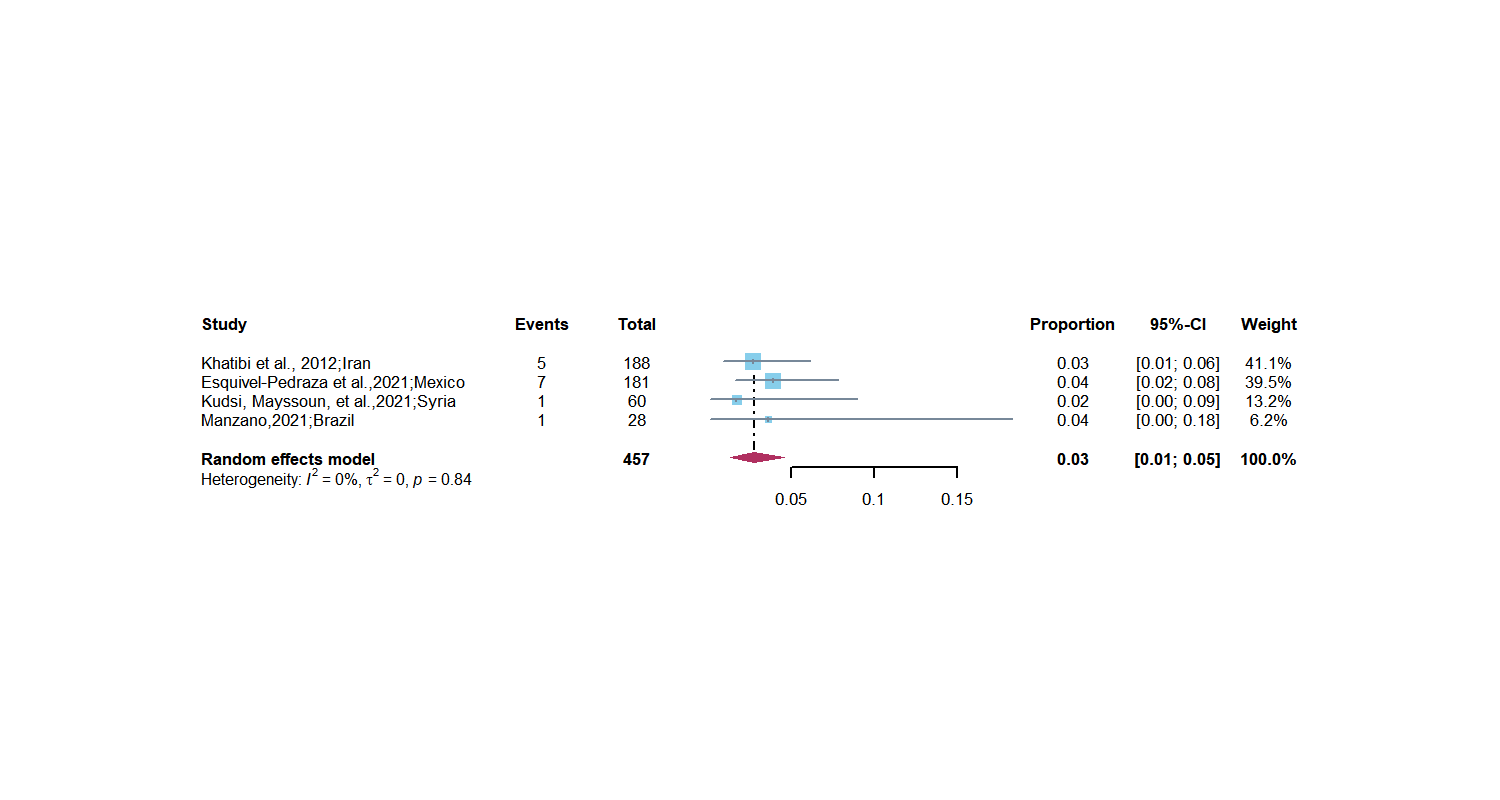


**Figure** **S13.** Prevalence of white plaque among SLE patients

1. **Prevalence of** **oral candidiasis among SLE patients (Forest plot of the included studies). (Supplementary Figure S14)**


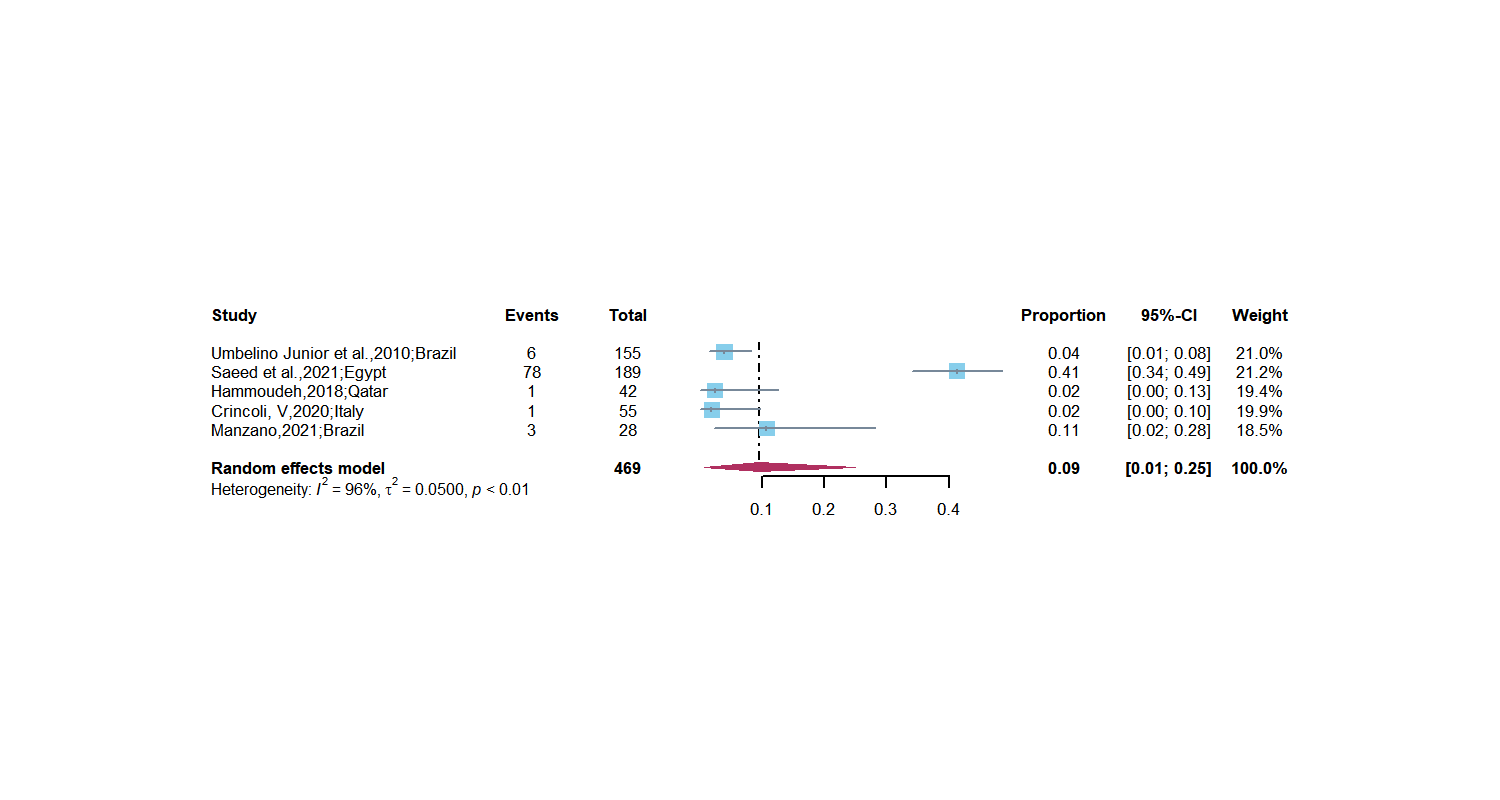


**Figure** **S14.** Prevalence of oral candidiasis among SLE patients

1. **Prevalence of petechiae among SLE patients (Forest plot of the included studies). (Supplementary Figure S15)**


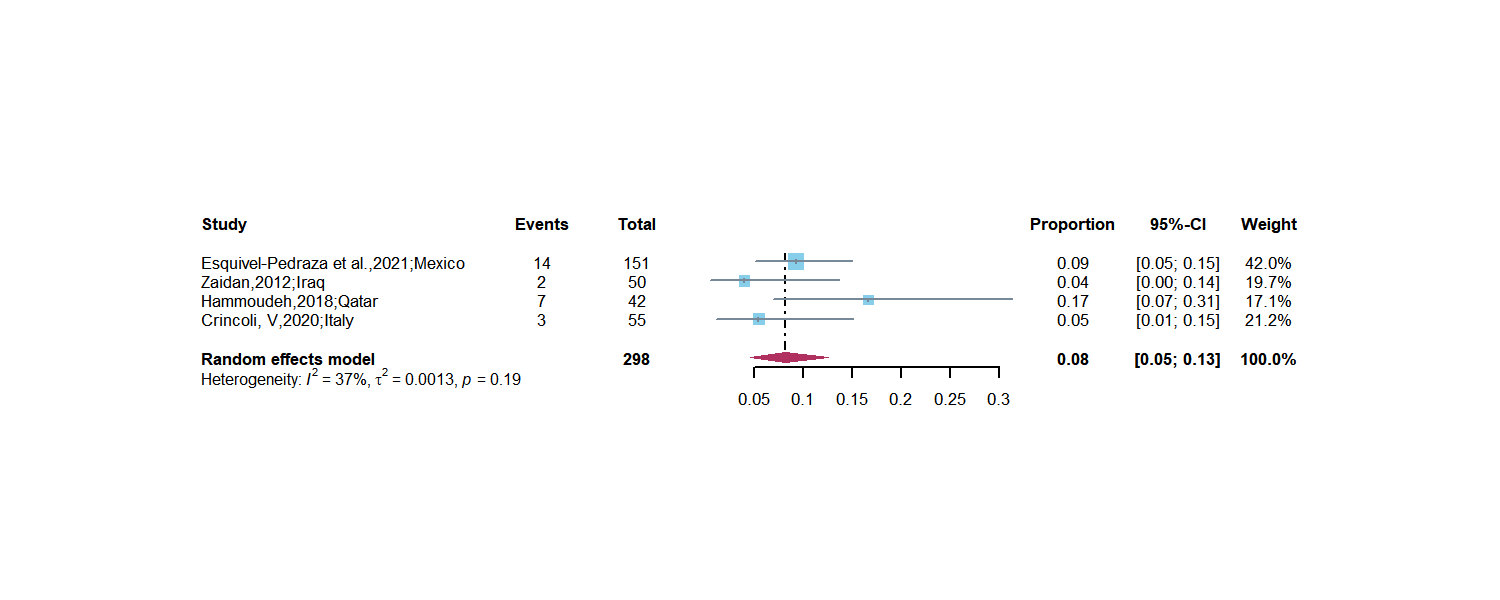


**Figure** **S15.** Prevalence of petechiae among SLE patients

1. **Prevalence of cheilitis among SLE patients (Forest plot of the included studies). (Supplementary Figure S16)**


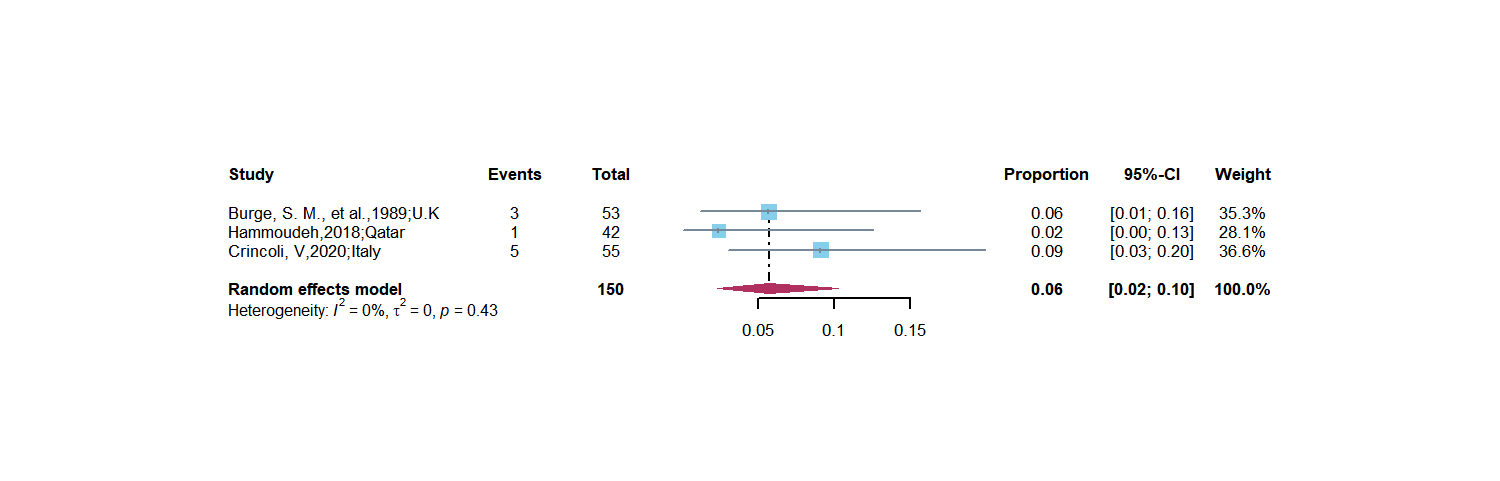


**Figure** **S16.** Prevalence of cheilitis among SLE patients

1. **Supplementary references (113 studies included in the meta-analysis):**

1. Aurlene N, Manipal S, Prabu D. Prevalence of oral mucosal lesions, dental caries, and periodontal disease among patients with systemic lupus erythematosus in a teaching hospital in Chennai, Tamil Nadu. J Family Med Prim Care. 2020;9:3374.

2. Khatibi M, Shakoorpour A, Jahromi ZM, Ahmadzadeh A. The prevalence of oral mucosal lesions and related factors in 188 patients with systemic lupus erythematosus. Lupus. 2012;21:1312-5.

3. Koh JH, Park EK, Lee HN, Kim Y, Kim GT, Suh YS, et al. Clinical characteristics and survival of 413 patients with systemic lupus erythematosus in southeastern areas of South Korea: a multicenter retrospective cohort study. Int J Rheum Dis. 2020;23:92-100.

4. Li Wg, Ye Zz, Yin Zh, Zhang K. Clinical and immunological characteristics in 552 systemic lupus erythematosus patients in a southern province of C hina. Int J Rheum Dis. 2017;20:68-75.

5. Zhao C, Zhao J, Huang Y, Wang Z, Wang H, Zhang H, et al. New-onset systemic lupus erythematosus during pregnancy. Clin Rheumatol. 2013;32:815-22.

6. Abd El Monem Teama M, Adham El-Mohamdy M, Abdellah Abdullah Mahmoud F, Mohammed Badr F. Autoantibody Profile of Egyptian Juvenile Systemic Lupus Erythematosus Patients and Its Association with Clinical Characteristics and Disease Activity. Open Access Rheumatol: Res Rev. 2021:201-12.

7. Meyer A, Guffroy A, Blaison G, Dieudonne Y, Amoura Z, Bonnotte B, et al. Systemic lupus erythematosus and neutropaenia: a hallmark of haematological manifestations. Lupus Sci Med. 2020;7:e000399.

8. Mao Y-M, Shi P-L, Wu L, Hu Y-Q, He Y-S, Xiang K, et al. Prevalence and influential factors of thrombocytopaenia in systemic lupus erythematosus patients: a retrospective analysis of 3140 cases in a Chinese population. Lupus. 2020;29:743-50.

9. Alonso MD, Martínez-Vázquez F, Riancho-Zarrabeitia L, Díaz de Terán T, Miranda-Filloy JA, Blanco R, et al. Sex differences in patients with systemic lupus erythematosus from Northwest Spain. Rheumatol Int. 2014;34:11-24.

10. To C, Mok C, Tang S, Ying S, Wong R, Lau C. Prognostically distinct clinical patterns of systemic lupus erythematosus identified by cluster analysis. Lupus. 2009;18:1267-75.

11. Wang W, Gao H. The combined detection of autoantibody characteristics in systemic lupus erythematosus. Am J Transl Res. 2021;13:7242.

12. Tejera Segura B, Altabás González I, Rúa-Figueroa I, Pérez Veiga N, Del Campo Pérez V, Olivé-Marqués A, et al. Relevance of gastrointestinal manifestations in a large Spanish cohort of patients with systemic lupus erythematosus: what do we know? Rheumatology. 2021;60:5329-36.

13. Soltani Z, Baghdadi A, Nejadhosseinian M, Faezi ST, Shahbazkhani B, Mousavi SA, et al. Celiac disease in patients with systemic lupus erythematosus. Reumatologia/Rheumatology. 2021;59:85-9.

14. Sieiro Santos C, Moriano Morales C, Álvarez Castro C, Díez Alvarez E. Polyautoimmunität bei systemischem Lupus erythematosus: sekundäres Sjögren-Syndrom. Z Rheumatol. 2021:1-6.

15. Lu W, Zhong Y, Zhang Y, Liu Z, Xue L. The Clinical Characteristics of Leukopenia in Patients with Systemic Lupus Erythematosus of Han Ethnicity in China: A Cross-Sectional Study. Rheumatol Ther. 2021;8:1177-88.

16. Costa Pires T, Caparrós-Ruiz R, Gaspar P, Isenberg DA. Prevalence and outcome of thrombocytopenia in systemic lupus erythematous: single-centre cohort analysis. Clin Exp Rheumatol. 2020.

17. Mayorga PAQ, Mesa JAR, Gualteros CCG, Benjumea PAP. Clinical and serological predictors of neuropsychiatric manifestations in patients with systemic lupus erythematosus. Rev Colomb Reumatol. 2023;30:214-21.

18. Othmani S, Louzir B. Systemic lupus erythematosus in 24 Tunisian males: clinical, laboratory and evolution analysis. Rev Med Interne. 2002;23:983-90.

19. Mok C, Tang S, To C, Petri M. Incidence and risk factors of venous thromboembolism in systemic lupus erythematosus: A comparison of 3 ethnic groups. Ann Rheum Dis. 2005;64:242-.

20. Wang J, Yang S, Chen J, Zhou S, He S, Liang Y, et al. Systemic lupus erythematosus: a genetic epidemiology study of 695 patients from China. Arch Dermatol Res. 2007;298:485-91.

21. AlSaleh J, Jassim V, ElSayed M, Saleh N, Harb D. Clinical and immunological manifestations in 151 SLE patients living in Dubai. Lupus. 2008;17:62-6.

22. Mongkoltanatus J, Wangkaew S, Kasitanon N, Louthrenoo W. Clinical features of Thai male lupus: an age-matched controlled study. Rheumatol Int. 2008;28:339-44.

23. Nazarinia M, Ghaffarpasand F, Shamsdin A, Karimi A, Abbasi N, Amiri A. Systemic lupus erythematosus in the Fars Province of Iran. Lupus. 2008;17:221-7.

24. Ramírez Gómez L, Uribe Uribe O, Osio Uribe O, Grisales Romero H, Cardiel M, Wojdyla D, et al. Childhood systemic lupus erythematosus in Latin America. The GLADEL experience in 230 children. Lupus. 2008;17:596-604.

25. Al Arfaj A, Khalil N. Clinical and immunological manifestations in 624 SLE patients in Saudi Arabia. Lupus. 2009;18:465-73.

26. Kole AK, Ghosh A. Cutaneous manifestations of systemic lupus erythematosus in a tertiary referral center. Indian J Dermatol. 2009;54:132.

27. Mantovani APF, Monclaro MP, Skare TL. Prevalence of IgA deficiency in adult systemic lupus erythematosus and the study of the association with its clinical and autoantibody profiles. Rev Bras Reumatol. 2010;50:273-82.

28. Umbelino Júnior AA, Cantisano MH, Klumb EM, Dias EP, Silva AAd. Oral and laboratorial findings in patients with systemic lupus erythematosus. J Bras Patol Med Lab. 2010;46:479-86.

29. Alonso-Perez E, Suarez-Gestal M, Calaza M, Witte T, Papasteriades C, Marchini M, et al. Association of systemic lupus erythematosus clinical features with European population genetic substructure. PLoS One. 2011;6:e29033.

30. Jönsen A, Clarke A, Joseph L, Belisle P, Bernatsky S, Nived O, et al. Association of the Charlson comorbidity index with mortality in systemic lupus erythematosus. Arthritis Care Res. 2011;63:1233-7.

31. Stefanidou S, Benos A, Galanopoulou V, Chatziyannis I, Kanakoudi F, Aslanidis S, et al. Clinical expression and morbidity of systemic lupus erythematosus during a post-diagnostic 5-year follow-up: a male: female comparison. Lupus. 2011;20:1090-4.

32. Weckerle CE, Franek BS, Kelly JA, Kumabe M, Mikolaitis RA, Green SL, et al. Network analysis of associations between serum interferon‐α activity, autoantibodies, and clinical features in systemic lupus erythematosus. Arthritis Rheumatol. 2011;63:1044-53.

33. Pettersson S, Lövgren M, Eriksson LE, Moberg C, Svenungsson E, Gunnarsson I, et al. An exploration of patient-reported symptoms in systemic lupus erythematosus and the relationship to health-related quality of life. Scand J Rheumatol. 2012;41:383-90.

34. Sánchez E, Rasmussen A, Riba L, Acevedo‐Vasquez E, Kelly JA, Langefeld CD, et al. Impact of genetic ancestry and sociodemographic status on the clinical expression of systemic lupus erythematosus in American Indian–European populations. Arthritis Rheumatol. 2012;64:3687-94.

35. Lee P, Yeh K, Yao T, Lee W, Lin Y, Huang J. The outcome of patients with renal involvement in pediatric-onset systemic lupus erythematosus–a 20-year experience in Asia. Lupus. 2013;22:1534-40.

36. Li PH, Wong WHS, Lee TL, Lau CS, Chan TM, Leung AMH, et al. Relationship between autoantibody clustering and clinical subsets in SLE: cluster and association analyses in Hong Kong Chinese. Rheumatology. 2013;52:337-45.

37. Fredi M, Cavazzana I, Quinzanini M, Taraborelli M, Cartella S, Tincani A, et al. Rare autoantibodies to cellular antigens in systemic lupus erythematosus. Lupus. 2014;23:672-7.

38. Li J, Leng X, Li Z, Ye Z, Li C, Li X, et al. Chinese SLE treatment and research group registry: III. association of autoantibodies with clinical manifestations in Chinese patients with systemic lupus erythematosus. J Immunol Res. 2014;2014.

39. Teh C, Ling G, Aishah WS. The Sarawak lupus cohort: clinical features and disease patterns of 633 SLE patients in a single tertiary centre from East Malaysia. Rheumatol Int. 2015;35:153-7.

40. Fakhreldin S, Gamal SM, Saad AS. Predictive potential of the disease activity index and C-reactive protein for infection in systemic lupus erythematosus patients. Egypt Rheumatol. 2015;37:171-5.

41. Suszek D, Majdan M. Clinical picture of late-onset systemic lupus erythematosus in a group of Polish patients. Polskie Arch Med Wewnetrznej. 2015;125.

42. Joo YB, Bae SC. Assessment of clinical manifestations, disease activity and organ damage in 996 Korean patients with systemic lupus erythematosus: comparison with other Asian populations. Int J Rheum Dis. 2015;18:117-28.

43. Penaranda-Parada E, Quintana G, Yunis J, Mantilla R, Rojas W, Panqueva U, et al. Clinical, serologic, and immunogenetic characterization (HLA-DRB1) of late-onset lupus erythematosus in a Colombian population. Lupus. 2015;24:1293-9.

44. Ambrose N, Morgan T, Galloway J, Ionnoau Y, Beresford M, Isenberg D. Differences in disease phenotype and severity in SLE across age groups. Lupus. 2016;25:1542-50.

45. Rasheed A, Rasul S, Hameed A. Prevalence of usual and unusual skin manifestations of systemic lupus erythematosus in a tertiary care hospital. J Pak Assoc Dermatol. 2016;26:118-22.

46. Artim-Esen B, Şahin S, Çene E, Şahinkaya Y, Barut K, Adrovic A, et al. Comparison of disease characteristics, organ damage, and survival in patients with juvenile-onset and adult-onset systemic lupus erythematosus in a combined cohort from 2 tertiary centers in Turkey. J Rheumatol. 2017;44:619-25.

47. Budhoo A, Mody G, Dubula T, Patel N, Mody P. Comparison of ethnicity, gender, age of onset and outcome in South Africans with systemic lupus erythematosus. Lupus. 2017;26:438-46.

48. Maloney K, Ferguson T, Stewart H, Myers A, De Ceulaer K. Clinical and immunological characteristics of 150 systemic lupus erythematosus patients in Jamaica: a comparative analysis. Lupus. 2017;26:1448-56.

49. Morais SA, Isenberg DA. A study of the influence of ethnicity on serology and clinical features in lupus. Lupus. 2017;26:17-26.

50. Sassi RH, Hendler JV, Piccoli GF, Gasparin AA, da Silva Chakr RM, Brenol JCT, et al. Age of onset influences on clinical and laboratory profile of patients with systemic lupus erythematosus. Clin Rheumatol. 2017;36:89-95.

51. Aggarwal A, Phatak S, Srivastava P, Lawrence A, Agarwal V, Misra R. Outcomes in juvenile onset lupus: single center cohort from a developing country. Lupus. 2018;27:1867-75.

52. Al Dowaiki S, Siddiqi AA, Al Kaabi J, Siddiqui UA, Khan MAU. Demographic, clinical and immunological manifestations of systemic lupus erythematosus among Omani population single tertiary care experience. Liaquat Univ Med Health Sci. 2018;17:208-14.

53. Fedrigo A, Dos Santos T, Nisihara R, Skare T. The lupus patient with positive rheumatoid factor. Lupus. 2018;27:1368-73.

54. Fonseca R, Aguiar F, Rodrigues M, Brito I. Clinical phenotype and outcome in lupus according to age: a comparison between juvenile and adult onset. Reumatol Clin. 2018;14:160-3.

55. Mahmoud G, Shahin A, Zayed H, Moghazy A, Eissa B. Clinical and immunological pattern and outcome of Egyptian systemic lupus erythematosus patients: a single center experience. Lupus. 2018;27:1562-9.

56. Manzano‐Gamero V, Pardo‐Cabello AJ, Vargas‐Hitos JA, Zamora‐Pasadas M, Navarrete‐Navarrete N, Sabio JM, et al. Effect of ethnicity on clinical presentation and risk of antiphospholipid syndrome in Roma and Caucasian patients with systemic lupus erythematosus: a multicenter cross‐sectional study. Int J Rheum Dis. 2018;21:2028-35.

57. Wei S, Yang Z, Xie S, Peng X, Gong L, Zhao K, et al. Autoimmune thyroid disease in patients with systemic lupus erythematosus: a 7-year retrospective study in China. Am J Med Sci. 2018;356:344-9.

58. Xiangfang C, Wenzhong Q, Zheng X, Shiwen Z, Pin L, Xuwei Y. Clinical features and risk factors of pulmonary hypertension in Chinese patients with systemic lupus erythematosus. Arch Rheumatol. 2019;34:88.

59. Gergianaki I, Fanouriakis A, Adamichou C, Spyrou G, Mihalopoulos N, Kazadzis S, et al. Is systemic lupus erythematosus different in urban versus rural living environment? Data from the Cretan Lupus Epidemiology and Surveillance Registry. Lupus. 2019;28:104-13.

60. Hamijoyo L, Candrianita S, Rahmadi A, Dewi S, Darmawan G, Suryajaya B, et al. The clinical characteristics of systemic lupus erythematosus patients in Indonesia: a cohort registry from an Indonesia-based tertiary referral hospital. Lupus. 2019;28:1604-9.

61. Ahn SS, Yoo J, Jung SM, Song JJ, Park Y-B, Lee S-W. Comparison of clinical features and outcomes between patients with early and delayed lupus nephritis. BMC Nephrol. 2020;21:1-9.

62. Saeed HM, Amr EM, Rezk ARL, Abd Elmoneim W. Prevalence of oral manifestations in patients with lupus erythematosus in a sample of the Egyptian population: a hospital based cross-sectional study. F1000Res. 2021;10.

63. Chebbi D, Jallouli M, Snoussi M, Damak C, Frikha F, Ben Salah R, et al. Familial lupus in Tunisia: a series of 14 families. Lupus. 2020;29:92-5.

64. Lim SC, Chan EWL, Tang SP. Clinical features, disease activity and outcomes of Malaysian children with paediatric systemic lupus erythematosus: A cohort from a tertiary centre. Lupus. 2020;29:1106-14.

65. Nikolopoulos D, Kostopoulou M, Pieta A, Karageorgas T, Tseronis D, Chavatza K, et al. Evolving phenotype of systemic lupus erythematosus in Caucasians: low incidence of lupus nephritis, high burden of neuropsychiatric disease and increased rates of late-onset lupus in the ‘Attikon’cohort. Lupus. 2020;29:514-22.

66. Prevete I, Espinosa G, Bellisai F, Bortoluzzi A, Conti F, Fredi M, et al. Comparative study between two European inception cohorts of patients with early systemic lupus erythematosus. Clin Exp Rheumatol. 2020;38:925-32.

67. Talukdar D, Gogoi AP, Doley D, Marak RR, Kakati S, Pradhan V, et al. The clinical and immunological profiles of systemic lupus erythematosus patients from Assam, North-East India. Indian J Rheumatol. 2020;15:181-6.

68. Chanprapaph K, Tubtieng I, Pratumchat N, Thadanipon K, Rattanakaemakorn P, Suchonwanit P. Cutaneous, systemic features and laboratory characteristics of late-versus adult-onset systemic lupus erythematosus in 1006 Thai patients. Lupus. 2021;30:785-94.

69. Esquivel-Pedraza L, Fernández-Cuevas L, Cicero-Casarrubias A, de León-Rosales SP, Fernández-Sánchez M, Orozco-Topete R, et al. Clinical characteristics of oral mucosal lesions in patients with systemic lupus erythematosus and their association with clinical and laboratory parameters. GSC Adv Res Rev. 2021;6:001-12.

70. Cervera R, Khamashta M, Hughes G. The Euro-lupus project: epidemiology of systemic lupus erythematosus in Europe. Lupus. 2009;18:869-74.

71. Correa-Rodríguez M, Pocovi-Gerardino G, Callejas-Rubio JL, Ríos-Fernández R, Martín-Amada M, Cruz-Caparrós M-G, et al. Clinical and serological associations of autoantibodies in patients with systemic lupus erythematosus. J Investig Med. 2021;69:1417-25.

72. Chottawornsak N, Rodsaward P, Suwannachote S, Rachayon M, Rattananupong T, Deekajorndech T, et al. Skin signs in juvenile-and adult-onset systemic lupus erythematosus: clues to different systemic involvement. Lupus. 2018;27:2069-75.

73. Uthman I, Nasr F, Kassak K, Masri A-F. Systemic lupus erythematosus in Lebanon. Lupus. 1999;8:713-5.

74. Ramírez Sepúlveda JI, Bolin K, Mofors J, Leonard D, Svenungsson E, Jönsen A, et al. Sex differences in clinical presentation of systemic lupus erythematosus. Biol Sex Differ. 2019;10:1-7.

75. Jasmin R, Sockalingam S, Cheah T, Goh K. Systemic lupus erythematosus in the multiethnic Malaysian population: disease expression and ethnic differences revisited. Lupus. 2013;22:967-71.

76. Aleem A, Al Arfaj AS, Khalil N, Alarfaj H. Haematological abnormalities in systemic lupus erythematosus. Acta Reumatol Port. 2014;39:236-41.

77. De Oliveira NT, Silva NG, Dos Santos TAG, Nisihara R, Skare TL. Clinical and autoantibody profile in male and female patients with systemic lupus erythematosus: a retrospective study in 603 Brazilian patients. Eur J Rheumatol. 2020;7:164.

78. Alballa S. Systemic lupus erythematosus in Saudi patients. Clin Rheumatol. 1995;14:342-6.

79. Abid N, Khan AS, Otaibi FHA. Systemic lupus erythematosus (SLE) in the eastern region of Saudi Arabia. A comparative study. Lupus. 2013;22:1529-33.

80. Novak G, Molinari B, Ferreira J, Sakamoto A, Terreri M, Pereira R, et al. Characteristics of 1555 childhood-onset lupus in three groups based on distinct time intervals to disease diagnosis: a Brazilian multicenter study. Lupus. 2018;27:1712-7.

81. QAMAR S, NAZ S, BATOOL S, REHMAN R, MUSHTAQ A, ALVI MY, et al. Clinical and Immunological Profile of Childhood Systemic Lupus Erythematosus: Observational Study. Pak J Med Health Sci. 2022;16:129-30.

82. AL-RAWI Z, Al-Shaarbaf H, Al-Raheem E, Khalifa S. Clinical features of early cases of systemic lupus erythematosus in Iraqi patients. Rheumatology (Oxford). 1983;22:165-71.

83. Al-Jarallah K, Al-Awadi A, Siddiqui H, Al-Salim I, Shehab D, Umamaheswaran I, et al. Systemic lupus erythematosus in Kuwait—hospital based study. Lupus. 1998;7:434-8.

84. Antolin J, Amerigo M, Cantabrana A, Roces A, Jimenez P. Systemic lupus erythematosus: clinical manifestations and immunological parameters in 194 patients. Subgroup classification of SLE. Clin Rheumatol. 1995;14:678-85.

85. Burge S, Frith P, Juniper R, Wojnarowska F. Mucosal involvement in systemic and chronic cutaneous lupus erythematosus. British Journal of Dermatology. 1989;121:727-41.

86. Mumtaz S, Ali M, Khan NB, Mehmood B, Azhar M, Farhan F. ORAL ULCERS IN SYSTEMIC LUPUS ERYTHEMATOSUS–RELATIONSHIP WITH DISEASE DURATION AND SEVERITY. J Khyber Coll Dent. 2020;10:121-5.

87. Johnson A, Cavalcanti F, Gordon C, Nived O, Palmer R, Sturfelt G, et al. Cross-sectional analysis of the differences between patients with systemic lupus erythematosus in England, Brazil and Sweden. Lupus. 1994;3:501-6.

88. Kudsi M, Nahas LD, Alsawah R, Hamsho A, Omar A. The prevalence of oral mucosal lesions and related factors in systemic lupus erythematosus patients. Arthritis Res Ther. 2021;23:1-5.

89. Tomic-Lucic A, Petrovic R, Radak-Perovic M, Milovanovic D, Milovanovic J, Zivanovic S, et al. Late-onset systemic lupus erythematosus: clinical features, course, and prognosis. Clin Rheumatol. 2013;32:1053-8.

90. Pradhan V, Patwardhan M, Nadkarni A, Ghosh K. Fc γ R IIB gene polymorphisms in Indian systemic lupus erythematosus (SLE) patients. Indian J Med Res. 2011;134:181.

91. Wang H, Gao Q, Liao G, Ren S, You W. Clinico-Laboratory Features and Associated Factors of Lupus Mesenteric Vasculitis. Rheumatol Ther. 2021;8:1031-42.

92. Meyer U, Kleinheinz J, Handschel J, Kruse‐Lösler B, Weingart D, Joos U. Oral findings in three different groups of immunocompromised patients. J Oral Pathol Med. 2000;29:153-8.

93. Segasothy M, Phillips PA. Systemic lupus erythematosus in Aborigines and Caucasians in central Australia: a comparative study. Lupus. 2001;10:439-44.

94. Feng H, Zhang G, Xie H, Chen M, Shi W, Wang L. Hepatitis C virus infection combined with systemic lupus erythematosus. J Cent South Univ (Med Sci). 2006;31:891-3.

95. Chen T-L, Wong C-H, Lee C-S, Loo J-H, Lin M. Systemic lupus erythematosus in the elderly. Int J Gerontol. 2009;3:108-13.

96. Shabana AA, El-Ghawet AE, Machaly SA, Abu Hashim EM, El-Kady BA, Shaat R. Anti-chromatin and anti-histone antibodies in Egyptian patients with systemic lupus erythematosus. Clin Rheumatol. 2009;28:673-8.

97. Fernandes EGC, Guissa VR, Saviolli C, Siqueira JTT, Valente M, Silva CAAd. Osteonecrosis of the jaw on imaging exams of patients with juvenile systemic lupus erythematosus. Rev Bras Reumatol. 2010;50:3-15.

98. Gonçalves LM, Bezerra Júnior JRS, Cruz MCFNd. Clinical evaluation of oral lesions associated with dermatologic diseases. An Bras Dermatol. 2010;85:150-6.

99. BAŞTUĞ F, POYRAZOĞLU H, Gündüz Z, Tülpar S, DÜŞÜNSEL R. Juvenile lupus erythematosus: fourteen years of experience. Arch Rheumatol. 2011;26:308-15.

100. Nakashima CAK, Galhardo AP, Silva JFMd, Fiorenzano GR, Santos ABdSd, Leite MFS, et al. Incidence and clinical-laboratory aspects of systemic lupus erythematosus in a Southern Brazilian city. Rev Bras Reumatol. 2011;51:235-9.

101. Dönmez S, Pamuk ÖN, Ümit EG, Top MŞ. Autoimmune rheumatic disease associated symptoms in fibromyalgia patients and their influence on anxiety, depression and somatisation: a comparative study. Clinical and experimental rheumatology. 2012;30:65-9.

102. Saigal R, Kansal A, Mittal M, Singh Y, Maharia HR, Juneja M. Clinical profile of systemic lupus erythematosus patients at a tertiary care centre in Western India. J Indian Acad Clin Med. 2011;13:27-32.

103. Zaidan TF. Oral manifestations, oral health status and saliva composition changes in a sample of Iraqi systemic lupus erythematosus patients. J Baghdad Coll Dent. 2012:65.

104. Zakeri Z, Narouie B, Bakhshipour A, Sarabadani J. Prevalence of oral manifestations in patient with systemic lupus erythematosus (SLE). Life Sci J. 2012;9:1307-11.

105. Tareen A, Naqi N, Afzal A, Malik U. Diagnostic accuracy of antinuclear antibodies and anti-double stranded DNA antibodies in patients of systemic lupus erythematosus presenting with dermatological features. J Pak Assoc Dermatol. 2014;24:127-31.

106. Compagno M, Gullstrand B, Jacobsen S, Eilertsen GØ, Nilsson JÅ, Lood C, et al. The assessment of serum-mediated phagocytosis of necrotic material by polymorphonuclear leukocytes to diagnose and predict the clinical features of systemic lupus erythematosus: an observational longitudinal study. Arthritis Res Ther. 2016;18:1-11.

107. Ahadian H, Ardakani T, Zahmati A, Owlia M, Zokaee H. Oral and Temporomandibular Joint Signs and Symptoms in New Cases of Autoimmune Connective-Tissue Diseases before the Treatment Onset. Ann Dent Spec. 2017;5:146-50.

108. Hammoudeh M, Al-Momani A, Sarakbi H, Chandra P, Hammoudeh S. Oral Manifestations of systemic lupus erythematosus patients in Qatar: A pilot study. Int J Rheumatol. 2018;2018.

109. Crincoli V, Piancino MG, Iannone F, Errede M, Di Comite M. Temporomandibular disorders and oral features in systemic lupus erythematosus patients: An observational study of symptoms and signs. Int J Med Sci. 2020;17:153.

110. Kumar P, Bharath G, Soneja M, Ranjan P, Biswas A, Wig N. Spectrum of Infection among Admitted Systemic Lupus Erythematosus Patients. J Indian Acad Clin Med. 2020;21:25-8.

111. Bongomin F, Sekimpi M, Kaddumukasa M. Clinical and immunological characteristics of 56 patients with systemic lupus erythematosus in Uganda. Rheumatol Adv Pract. 2020;4:rkaa011.

112. Buonavoglia A, Leone P, Prete M, Solimando AG, Guastadisegno C, Lanave G, et al. Epstein–Barr Virus in Salivary Samples from Systemic Lupus Erythematosus Patients with Oral Lesions. J Clin Med. 2021;10:4995.

113. Manzano BR, da Silva Santos PS, Bariquelo MH, Merlini NRG, Honório HM, Rubira CMF. A case-control study of oral diseases and quality of life in individuals with rheumatoid arthritis and systemic lupus erythematosus. Clin Oral Investig. 2021;25:2081-92.

| **Section and Topic** | **Item #** | **Checklist item** | **Location where item is reported** |
| --- | --- | --- | --- |
| **TITLE** | | |  |
| Title | 1 | Identify the report as a systematic review. | Page1,2 |
| **ABSTRACT** | | |  |
| Abstract | 2 | See the PRISMA 2020 for Abstracts checklist. | Page2 |
| **INTRODUCTION** | | |  |
| Rationale | 3 | Describe the rationale for the review in the context of existing knowledge. | Page3 |
| Objectives | 4 | Provide an explicit statement of the objective(s) or question(s) the review addresses. | Page3 |
| **METHODS** | | |  |
| Eligibility criteria | 5 | Specify the inclusion and exclusion criteria for the review and how studies were grouped for the syntheses. | Page3,4 |
| Information sources | 6 | Specify all databases, registers, websites, organisations, reference lists and other sources searched or consulted to identify studies. Specify the date when each source was last searched or consulted. | Page3 |
| Search strategy | 7 | Present the full search strategies for all databases, registers and websites, including any filters and limits used. | Page3,appendix |
| Selection process | 8 | Specify the methods used to decide whether a study met the inclusion criteria of the review, including how many reviewers screened each record and each report retrieved, whether they worked independently, and if applicable, details of automation tools used in the process. | Page4 |
| Data collection process | 9 | Specify the methods used to collect data from reports, including how many reviewers collected data from each report, whether they worked independently, any processes for obtaining or confirming data from study investigators, and if applicable, details of automation tools used in the process. | Page4 |
| Data items | 10a | List and define all outcomes for which data were sought. Specify whether all results that were compatible with each outcome domain in each study were sought (e.g. for all measures, time points, analyses), and if not, the methods used to decide which results to collect. | Page4 |
|  | 10b | List and define all other variables for which data were sought (e.g. participant and intervention characteristics, funding sources). Describe any assumptions made about any missing or unclear information. | Page4 |
| Study risk of bias assessment | 11 | Specify the methods used to assess risk of bias in the included studies, including details of the tool(s) used, how many reviewers assessed each study and whether they worked independently, and if applicable, details of automation tools used in the process. | Page4 |
| Effect measures | 12 | Specify for each outcome the effect measure(s) (e.g. risk ratio, mean difference) used in the synthesis or presentation of results. | Page4 |
| Synthesis methods | 13a | Describe the processes used to decide which studies were eligible for each synthesis (e.g. tabulating the study intervention characteristics and comparing against the planned groups for each synthesis (item #5)). | Page4 |
|  | 13b | Describe any methods required to prepare the data for presentation or synthesis, such as handling of missing summary statistics, or data conversions. | Page4 |
|  | 13c | Describe any methods used to tabulate or visually display results of individual studies and syntheses. | Page4 |
|  | 13d | Describe any methods used to synthesize results and provide a rationale for the choice(s). If meta-analysis was performed, describe the model(s), method(s) to identify the presence and extent of statistical heterogeneity, and software package(s) used. | Page4 |
|  | 13e | Describe any methods used to explore possible causes of heterogeneity among study results (e.g. subgroup analysis, meta-regression). | Page4 |
|  | 13f | Describe any sensitivity analyses conducted to assess robustness of the synthesized results. | Page4 |
| Reporting bias assessment | 14 | Describe any methods used to assess risk of bias due to missing results in a synthesis (arising from reporting biases). | Page4 |
| Certainty assessment | 15 | Describe any methods used to assess certainty (or confidence) in the body of evidence for an outcome. | Page4 |
| **RESULTS** | | |  |
| Study selection | 16a | Describe the results of the search and selection process, from the number of records identified in the search to the number of studies included in the review, ideally using a flow diagram. | Page4 |
|  | 16b | Cite studies that might appear to meet the inclusion criteria, but which were excluded, and explain why they were excluded. | Page4,5 |
| Study characteristics | 17 | Cite each included study and present its characteristics. | Page5 |
| Risk of bias in studies | 18 | Present assessments of risk of bias for each included study. | Page5 |
| Results of individual studies | 19 | For all outcomes, present, for each study: (a) summary statistics for each group (where appropriate) and (b) an effect estimate and its precision (e.g. confidence/credible interval), ideally using structured tables or plots. | Page4,5,appendix |
| Results of syntheses | 20a | For each synthesis, briefly summarise the characteristics and risk of bias among contributing studies. | Page5-7 |
|  | 20b | Present results of all statistical syntheses conducted. If meta-analysis was done, present for each the summary estimate and its precision (e.g. confidence/credible interval) and measures of statistical heterogeneity. If comparing groups, describe the direction of the effect. | Page5-7 |
|  | 20c | Present results of all investigations of possible causes of heterogeneity among study results. | Page5-7 |
|  | 20d | Present results of all sensitivity analyses conducted to assess the robustness of the synthesized results. | Page5-7 |
| Reporting biases | 21 | Present assessments of risk of bias due to missing results (arising from reporting biases) for each synthesis assessed. | Page5 |
| Certainty of evidence | 22 | Present assessments of certainty (or confidence) in the body of evidence for each outcome assessed. | N/A |
| **DISCUSSION** | | |  |
| Discussion | 23a | Provide a general interpretation of the results in the context of other evidence. | Page7 |
|  | 23b | Discuss any limitations of the evidence included in the review. | Page9 |
|  | 23c | Discuss any limitations of the review processes used. | Page9 |
|  | 23d | Discuss implications of the results for practice, policy, and future research. | Page9 |
| **OTHER INFORMATION** | | |  |
| Registration and protocol | 24a | Provide registration information for the review, including register name and registration number, or state that the review was not registered. | Page3 |
|  | 24b | Indicate where the review protocol can be accessed, or state that a protocol was not prepared. | Page3 |
|  | 24c | Describe and explain any amendments to information provided at registration or in the protocol. | Page3 |
| Support | 25 | Describe sources of financial or non-financial support for the review, and the role of the funders or sponsors in the review. | Page10 |
| Competing interests | 26 | Declare any competing interests of review authors. | Page10 |
| Availability of data, code and other materials | 27 | Report which of the following are publicly available and where they can be found: template data collection forms; data extracted from included studies; data used for all analyses; analytic code; any other materials used in the review. | N/A |

*From:*  Page MJ, McKenzie JE, Bossuyt PM, Boutron I, Hoffmann TC, Mulrow CD, et al. The PRISMA 2020 statement: an updated guideline for reporting systematic reviews. BMJ 2021;372:n71. doi: 10.1136/bmj.n71

For more information, visit: <http://www.prisma-statement.org/>
